# Citrobacter rodentium Infection Induces Persistent Molecular Changes and Interferon Gamma-Dependent Major Histocompatibility Complex Class II Expression in the Colonic Epithelium

**DOI:** 10.1128/mbio.03233-21

**Published:** 2022-02-01

**Authors:** Caroline Mullineaux-Sanders, Zuzanna Kozik, Julia Sanchez-Garrido, Eve G. D. Hopkins, Jyoti S. Choudhary, Gad Frankel

**Affiliations:** a Centre for Molecular Microbiology and Infection, Department of Life Sciences, Imperial College, London, United Kingdom; b Functional Proteomics Group, Chester Beatty Laboratories, Institute of Cancer Research, London, United Kingdom; University of Texas Southwestern Medical Center Dallas

**Keywords:** *Citrobacter rodentium*, enteric infection, antigen presentation

## Abstract

Most studies of infections at mucosal surfaces have focused on the acute phase of the disease. Consequently, little is known about the molecular processes that underpin tissue recovery and the long-term consequences postinfection. Here, we conducted temporal deep quantitative proteomic analysis of colonic intestinal epithelial cells (cIECs) from mice infected with the natural mouse pathogen Citrobacter rodentium over time points corresponding to the late steady-state phase (10 days postinfection [DPI]), the clearance phase (13 to 20 DPI), and 4 weeks after the pathogen has been cleared (48 DPI). *C. rodentium*, which relies on a type III secretion system to infect, is used to model infections with enteropathogenic and enterohemorrhagic *Escherichia coli*. We observe a strong upregulation of inflammatory signaling and nutritional immunity responses during the clearance phase of the infection. Despite morphological tissue recovery, chromogranin B (ChgB)-positive endocrine cells remained significantly below baseline levels at 48 DPI. In contrast, we observed an increased abundance of proteins involved in antigen processing and presentation 4 weeks after pathogen clearance. In particular, long-term changes were characterized by a persistent interferon gamma (IFN-γ) response and the expression of major histocompatibility complex class II (MHCII) molecules in 60% of the EpCAM^+^ cIECs, which were not seen in *Ifn*γ*^−/−^* mice. Nonetheless, both wild-type and *Ifn*γ*^−/−^* mice mounted similar systemic and colonic IgG responses to C. rodentium and were equally protected from rechallenge, suggesting that cIEC MHCII is not necessary for protective immunity against C. rodentium.

## INTRODUCTION

The intestinal epithelium is a proliferative mucosal barrier responsible for nutrient and water absorption (1, 2). Moreover, it provides a physical barrier to the microbiota and pathogens and orchestrates immune responses to infection (3). Colonic intestinal epithelial cells (cIECs), which are safeguarded by mucins secreted from goblet cells, express a number of pattern recognition receptors (PRRs) that enable the detection of pathogen-associated molecular patterns (PAMPs) and the activation of innate immune responses (4). Via interleukin-22 (IL-22) and interferon gamma (IFN-γ) receptors, cIECs respond to inflammatory signals through the induction of bacterial defense and epithelial regeneration pathways (5). The inability to restore gut barrier integrity following perturbation generates a “leaky gut,” which allows an anomalous interaction of the luminal contents with the intestinal mucosa and results in pathology (6).

The cIEC monolayer develops from LGR5-positive (LGR5^+^) stem cells at the base of the crypt, which give rise to partially differentiated transit-amplifying (TA) cells that fully differentiate into diverse cell types, including absorptive enterocytes, mucus-producing goblet cells, and hormone-producing enteroendocrine cells (7–9). Perturbations, including infection, antibiotic treatment, or severe dietary changes, can result in dysbiosis, rapid inflammatory responses, changes to the cellular composition of the crypt, and disruption of the mucosal barrier functions (10). It is thought that once the acute symptoms of these insults are resolved, the tissue returns to a preperturbation homeostatic state.

Citrobacter rodentium is a natural extracellular murine enteric pathogen that causes acute colitis while providing a robust model for the attaching and effacing (A/E) human pathogens enteropathogenic Escherichia coli (EPEC) and enterohemorrhagic E. coli (EHEC) (11). Infection with C. rodentium is dependent on the injection of effector proteins via a type III secretion system (T3SS), which manipulates multiple cell signaling pathways (12). For example, Tir mediates intimate bacterial attachment and activates NF-κB signaling (13), while NleC and NleE inhibit NF-κB signaling (14–16), and EspF and EspI/NleA alter tight junction integrity (17, 18).

C. rodentium infection of C57BL/6 mice results in mild and self-limiting disease; following oral inoculation, C. rodentium initially colonizes the cecal lymphoid patch from 1 to 3 days postinfection (DPI) (establishment phase) (19). At 4 DPI, C. rodentium sporadically colonizes the apex of the colonic crypts, followed by rapid expansion and extensive colonization of the colonic mucosa by 6 DPI (expansion phase) (20). C. rodentium shedding plateaus at approximately 10^9^ CFU/g stool at 8 to 12 DPI (steady-state phase) (21) before being spontaneously cleared, with typically undetectable levels of C. rodentium in the stool by 18 to 21 DPI (21).

Infected mice respond to C. rodentium infection by inducing colonic crypt hyperplasia (CCH), a tissue repair response characterized by the proliferation of TA cells, a loss of mature cell types, and a strong proinflammatory response. The proinflammatory cytokines IL-22 and IL-17 are produced from the early phases of infection (5, 22–24), and IL-22-deficient mice suffer from leaky gut, leading to systemic bacterial dissemination and fatality (5). Th1/17 cells produce IFN-γ, IL-21, IL-22, and IL-17 during the later stages of infection, with IFN-γ knockout (KO) mice presenting impaired clearance and increased colonic pathology compared to wild-type (WT) mice (25–27).

Our recent global proteomics analysis of cIECs during the establishment (4 and 6 DPI) and early peak (8 DPI) phases of infection uncovered changes to the immunometabolic axis and bioenergetics from the inception of C. rodentium colonic colonization (20, 28), including a switch from respiration via the citrate cycle (tricarboxylic acid [TCA] cycle) and oxidative phosphorylation (OXPHOS) to aerobic glycolysis (the Warburg effect). While studies of C. rodentium infection mainly focus on the expansion and steady-state phases, little is currently known about the molecular, cellular, and immunological changes in cIECs in the late steady-state and clearance phases (13 to 20 DPI) and beyond (i.e., weeks after the pathogen has cleared). Here, we use C. rodentium infection as a model to investigate temporal mucosal regeneration processes that arise following acute enteric infection and their roles in protective immunity.

## RESULTS

### Temporal cIEC proteomic responses to C. rodentium infection during the clearance and recovery phases.

**FIG 1:**
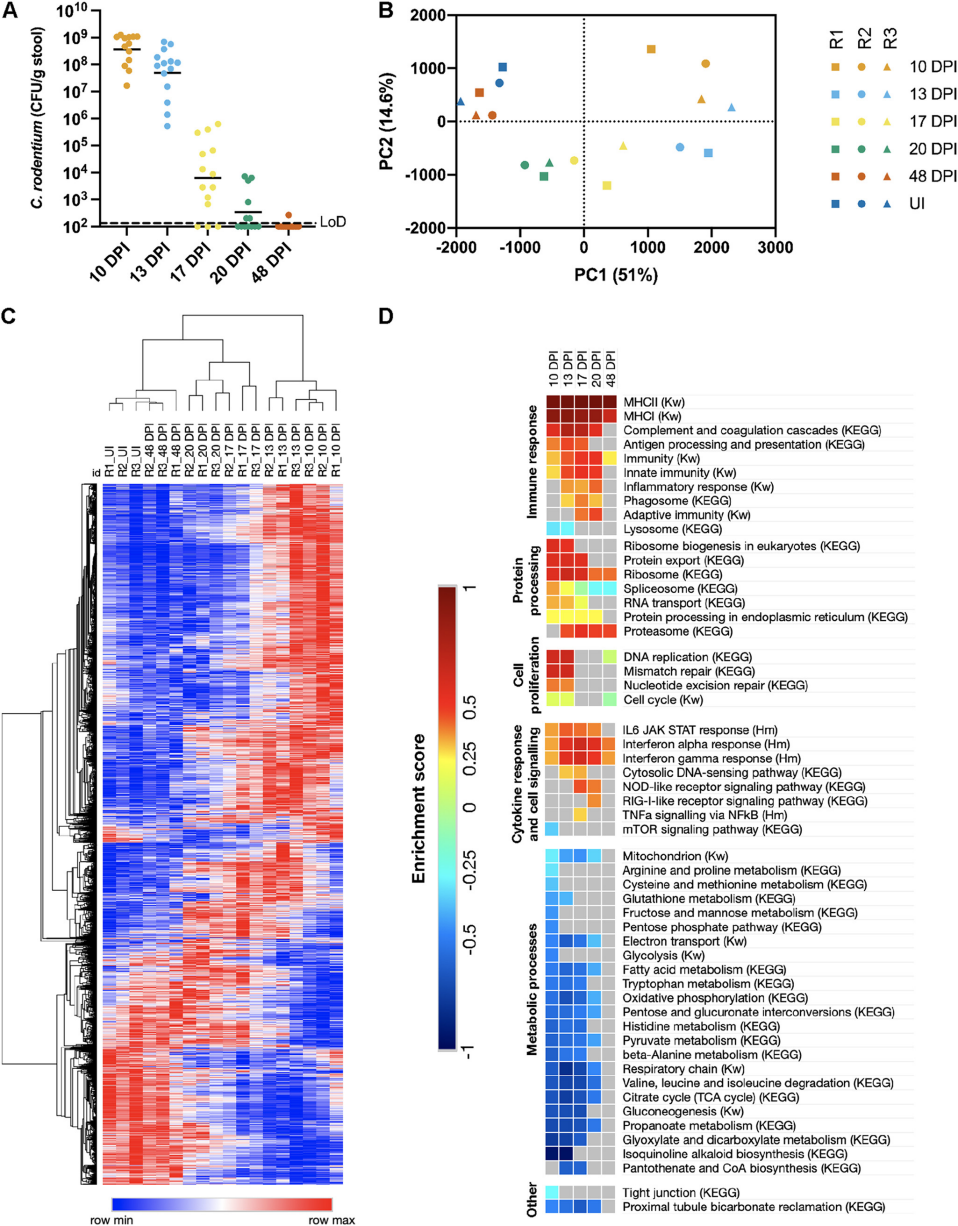
Temporal analysis of cIECs during the clearance and recovery phases of infection. (A) C. rodentium colonization of mice used for cIEC proteomic analysis. LoD, limit of detection; DPI, days postinfection. (B) PCA of the mouse protein relative abundance values. R, biological repeat. (C) Hierarchical clustering (one minus Pearson’s correlation) of relative abundance values of significantly changed mouse proteins (FDR of <0.05 by one-way ANOVA). (D) 1D enrichment scores of selected KEGG, Keywords (Kw), and Hallmarks (Hm) processes found to be significantly upregulated (positive enrichment score) or downregulated (negative enrichment score). The FDR is <0.02, with the exception of “cytosolic DNA sensing (KEGG),” where the FDR is <0.05. TNFa, tumor necrosis factor alpha.

We previously described cIEC responses to C. rodentium during the expansion phase (4 and 6 DPI) (20) and at the entry to the steady-state phase (8 DPI) (28) of infection. To characterize the temporal changes in cIECs during the late steady-state and clearance phases of the infection, we performed proteomic analysis of cIECs isolated from C57BL/6 mice at 10, 13, 17, and 20 DPI. Additionally, to characterize the nature and duration of the regeneration processes postinfection, we analyzed cIECs isolated 4 weeks after C. rodentium had been cleared (48 DPI). Mock-infected mice harvested at 10 DPI were used as uninfected (UI) controls. Analysis of enriched cIECs showed a purity of around 90 to 95% epithelial cell adhesion molecule-positive (EpCAM^+^) cells (see Fig. S1A in the supplemental material). As expected, C57BL/6 mice started to spontaneously clear C. rodentium after 10 DPI; the pathogen fell below the level of detection by immunofluorescence at 13 DPI, and the majority of mice no longer had detectable fecal C. rodentium by 20 DPI (Fig. 1A; Fig. S1B and C).

10.1128/mBio.03233-21.1 FIG S1 Temporal analysis of the clearance phase of C. rodentium infection (related to Fig. 1). (A) Quantification of EpCAM^+^ cells in cIEC-enriched preparations from uninfected and C. rodentium-infected mice. Each point represents an individual mouse. Data are from two biological repeats. (B) C. rodentium colonization of mice from which cIECs were extracted for proteomic analysis. LoD, limit of detection. (C) Representative images of immunofluorescence staining of C. rodentium in the distal colon, showing undetectable levels of mucosa-associated C. rodentium from 13 DPI. Red, C. rodentium; blue, DNA. Bar = 200 μm. Images are representative of results for at least 13 mice/group from 3 biological repeats. (D) Proteins significantly increased (“Up”) or decreased (“Down”) in abundance at each time point compared to UI mice. Significance was determined by Tukey’s HSD posttest (FDR of <0.05) performed on relative abundance values of proteins considered significantly changed by one-way ANOVA across all investigated time points (FDR of <0.05; 4,164 proteins). Download FIG S1, PDF file, 0.3 MB. Copyright © 2022 Mullineaux-Sanders et al. 2022 Mullineaux-Sanders et al. https://creativecommons.org/licenses/by/4.0/ This content is distributed under the terms of the Creative Commons Attribution 4.0 International license.

Three biological repeats for each time point (UI and 10, 13, 17, 20, and 48 DPI) were analyzed by liquid chromatography-tandem mass spectrometry (LC-MS/MS). We quantified a total of 8,439 unique proteins that mapped to the Mus musculus proteome (Table S1), 7,301 of which were identified in at least two biological repeats, and 814 unique proteins which mapped to the C. rodentium proteome (Table S2) (peptide false discovery rate [FDR] of <1%). The adhesin intimin and the T3SS effectors Tir, EspF, and EspI/NleA were highly enriched at 10 and 13 DPI before their abundance declined (Table S2).

10.1128/mBio.03233-21.6 TABLE S1 Mouse proteomic data. Log_2_FC values are displayed for mouse proteins detected in cIEC samples at the indicated days postinfection, normalized to the value for the UI sample from the same biological repeat. Three independent biological replicates (R1 to R3) are shown. The number of unique peptides, total number of identified peptide spectra matched for the protein (PSMs), and adjusted *P* values (*q* values) for each protein identified are also provided. Blank cells indicate that the protein was not detected in any sample in that TMT set. Download Table S1, XLSX file, 2.2 MB. Copyright © 2022 Mullineaux-Sanders et al. 2022 Mullineaux-Sanders et al. https://creativecommons.org/licenses/by/4.0/ This content is distributed under the terms of the Creative Commons Attribution 4.0 International license.

10.1128/mBio.03233-21.7 TABLE S2 C. rodentium proteomic data. Log_2_FC values are displayed for C. rodentium proteins detected in cIEC samples at the indicated days postinfection, normalized to the value for the UI sample from the same biological repeat. Three independent biological replicates (R1 to R3) are shown. The number of unique peptides, total number of identified peptide spectra matched for the protein (PSMs), and adjusted *P* values (*q* values) for each protein identified are also provided. Blank cells indicate that the protein was not detected in any sample in that TMT set. Download Table S2, XLSX file, 0.2 MB. Copyright © 2022 Mullineaux-Sanders et al. 2022 Mullineaux-Sanders et al. https://creativecommons.org/licenses/by/4.0/ This content is distributed under the terms of the Creative Commons Attribution 4.0 International license.

Principal-component analysis (PCA) of mouse proteins showed close clustering of biological replicates from UI and 48 DPI samples (Fig. 1B), demonstrating that most infection signatures in the proteome had returned to preinfection homeostasis by 48 DPI, 4 weeks after C. rodentium clearance at 20 DPI. One-way analysis of variance (ANOVA) identified 4,164 significantly changed proteins across all the investigated time points, and Tukey’s postanalysis (FDR of <0.05) showed that 10 and 13 DPI had the most significantly changed proteins (around 2,800) compared to UI mice (Fig. 1C; Fig. S1D). One-dimensional (1D) enrichment analysis showed temporal changes in cIEC metabolic and immune processes as they transitioned between phases of the infection cycle (Fig. 1D).

### The colonic crypt exhibits persistent changes 4 weeks after pathogen clearance.

**FIG 2:**
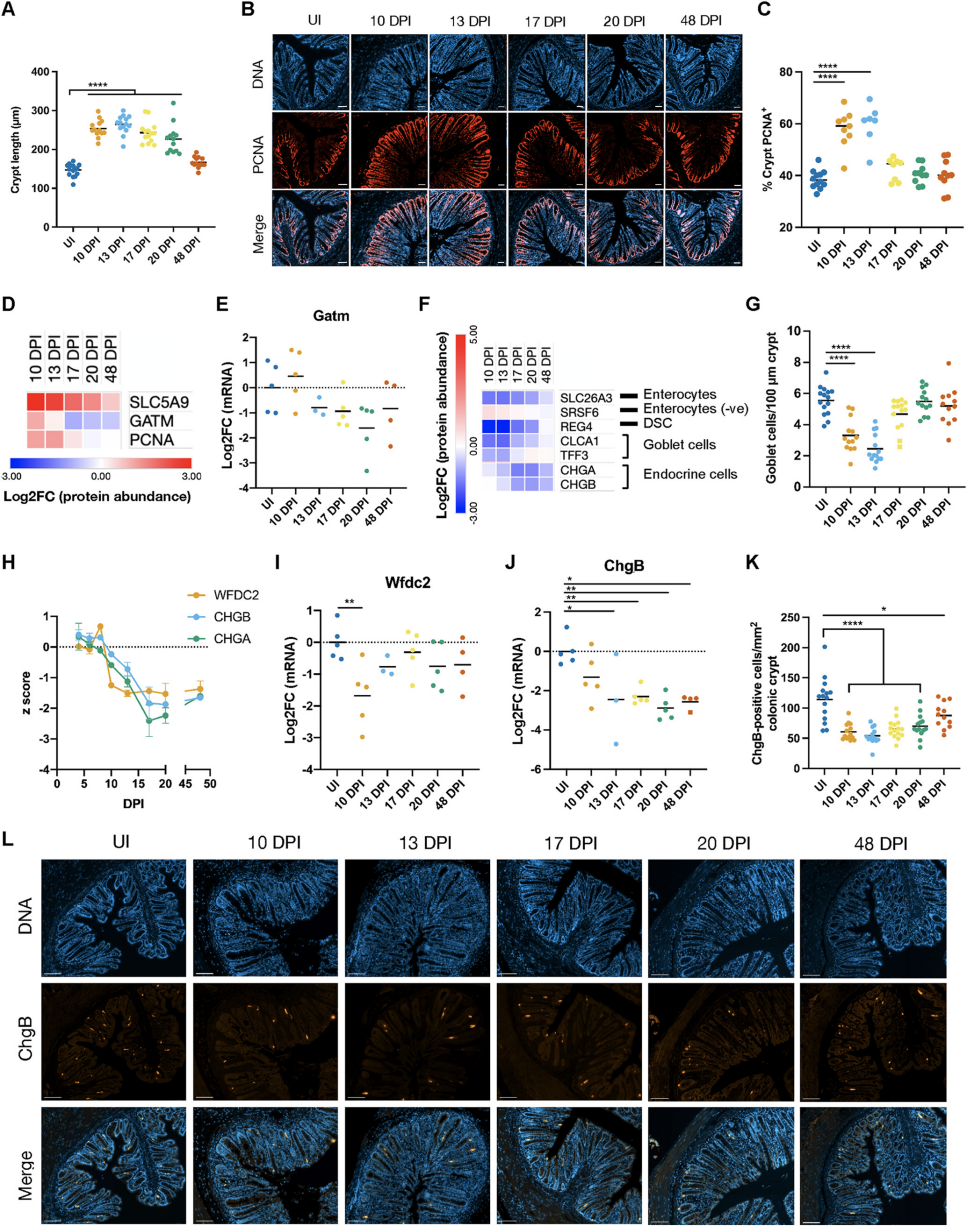
Changes to the cellular composition of the crypt. (A, C, G, and K) Temporal measurements of CCH (A), the crypt PCNA^+^ zone (C), goblet cell density (G), and CHGB-positive cell density (K). Each point represents the mean for an individual mouse. *, *P* < 0.0.5; ****, *P* < 0.0001 (by one-way ANOVA with Dunnett’s posttest for multiple comparisons to the control group [uninfected {UI} mice]). (B and L) Representative distal colonic sections showing temporal changes in the crypt PCNA^+^ zone (B) and CHGB-positive cell density (L). Bars = 50 μm (B) or 100 μm (L). (D and F) Average Log_2_FC values of selected significantly changed (FDR of <0.05 by one-way ANOVA) proteins involved in cell proliferation and cIEC metabolic changes (D) and cIEC markers (F). DSC, deep secretory cell. In panel F, “−ve” indicates that proteins are negative regulators of terminal differentiation into the specified cell type. (E, I, and J) qRT-PCR of the indicated mRNAs isolated from cIECs. Each point represents an individual mouse. *, *P* < 0.05; **, *P* < 0.01 (by one-way ANOVA with Dunnett’s posttest for multiple comparisons to the control group [uninfected mice]). For panels G and J, square data points were identified as outliers and not included in the statistical analysis. (H) z scores of selected protein abundances across the entire C. rodentium infection time course (4 to 48 DPI). Means ± standard errors of the means (SEM) are shown.

Consistent with C. rodentium-induced CCH, the cell proliferation processes “DNA replication,” “mismatch repair,” and “cell cycle” were significantly enriched at 10 to 13 DPI before recovering by 17 DPI (Fig. 1D). Curiously, proteins of the pentose phosphate pathway, the main source of nucleotide intermediates, were found in lower abundances at 4 to 10 DPI (20, 28) (Fig. 1D); therefore, cIECs seem to use alternative sources of nucleotides for cell proliferation. Both the expansion of the crypt length (CCH) (Fig. 2A; Fig. S2A) and the abundance of the cell proliferation marker PCNA peaked at 13 DPI before recovering, and immunofluorescence staining of PCNA mirrored and validated the proteomic changes seen in the cIECs (Fig. 2B to D).

10.1128/mBio.03233-21.2 FIG S2 Temporal measurement of CCH and goblet cell density (related to Fig. 2). Representative distal colonic images show CCH (A) and goblet cell density (B). Bars = 200 μm. Download FIG S2, PDF file, 0.6 MB. Copyright © 2022 Mullineaux-Sanders et al. 2022 Mullineaux-Sanders et al. https://creativecommons.org/licenses/by/4.0/ This content is distributed under the terms of the Creative Commons Attribution 4.0 International license.

The rapid cell proliferation of the epithelial barrier in response to infection is fueled by aerobic glycolysis (the Warburg effect) (20, 28). Consistently, mitochondrial processes involved in ATP production, including OXPHOS, fatty acid metabolism, and the TCA cycle, were inhibited during the clearance phase (10 to 20 DPI) and fully restored by 48 DPI (Fig. 1D). The basolateral glucose importer SLC5A9, which seems to fuel aerobic glycolysis, was highly upregulated at 10 and 13 DPI and remained modestly elevated at 48 DPI, while glycine amidinotransferase (GATM), which catalyzes creatine production for energy dispersal, was enriched at 10 DPI before being downregulated from 17 DPI onward (Fig. 2D and E).

CCH is associated with a loss of mature cIEC types, including absorptive enterocytes and goblet cells. Consistently, goblet cell (CLCA1 and TFF3) and deep secretory cell (REG4) markers were significantly reduced before beginning to recover to preinfection levels from 17 DPI onward (Fig. 2F). SLC26A3, a marker for differentiated enterocytes, was significantly decreased in abundance at all time points (although a trend toward recovery was observed from 20 DPI) (Fig. 2F). As previously reported (29), we observed a reduction in the density of alcian blue and periodic acid-Schiff (AB/PAS) stain (which stains all intracellular mucin glycoproteins)-positive cells at 10 to 13 DPI, after which it gradually returned to nearly UI levels, reflecting what was observed in the proteomics analysis (Fig. 2F and G; Fig. S2B). Interestingly, the goblet cell-secreted antimicrobial protein WFDC2, which plays an important role in preventing epithelial barrier breakdown (30), was present at a lower abundance from 10 to 48 DPI (Fig. 2H) and was transcriptionally downregulated specifically at 10 DPI (Fig. 2I). Together, these data show that despite a recovery of goblet cell density, these cells may remain functionally altered 4 weeks after pathogen clearance. Moreover, the abundance of the panendocrine cell markers chromogranin A (CHGA) and CHGB, which are weakly upregulated during the early stages of infection (Fig. 2F) (20), was decreased from 17 to 48 DPI (Fig. 2F), and *ChgB* was significantly transcriptionally downregulated from 13 to 48 DPI compared to UI mice (Fig. 2J). Staining of CHGB-positive cells in the colonic crypts showed a similar trend (Fig. 2K to L). Accordingly, C. rodentium-induced perturbation of the cellular composition of the colonic crypt fails to fully recover 4 weeks after infection clearance.

### Innate immune signaling pathways are upregulated during the clearance phase.

**FIG 3:**
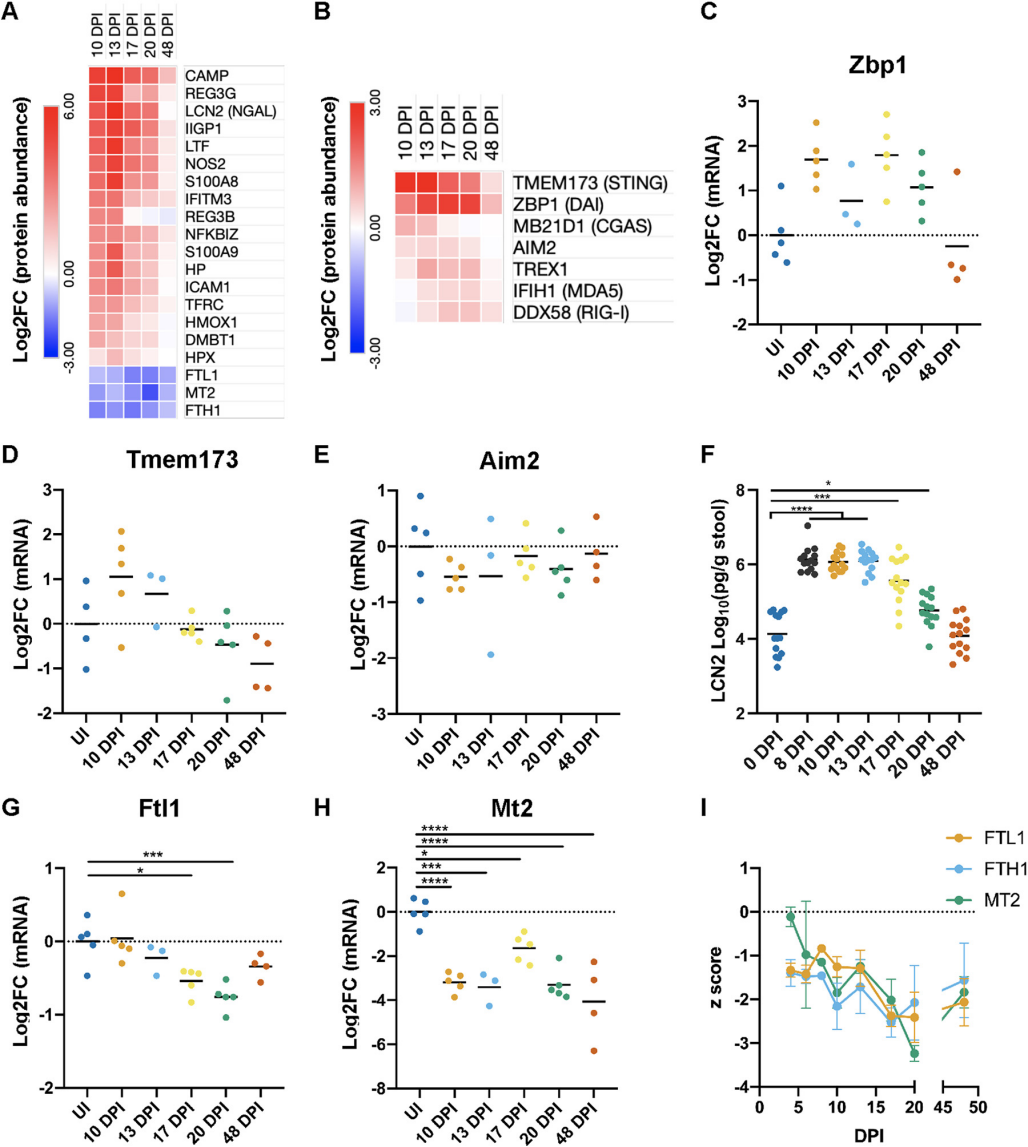
Temporal progression of innate immune proteins. (A and B) Average Log_2_FC values of selected innate immunity (A) and cytosolic nucleic acid-sensing (B) proteins. All proteins shown were significantly changed (FDR of <0.05 by one-way ANOVA), with the following exceptions: LCN-2 (*P* = 0.05004), S100A8 (*P* = 0.061), and S100A9 (*P* = 0.0513). (C to E, G, and H) qRT-PCR of the indicated mRNAs isolated from cIECs. Each point represents an individual mouse. *, *P* < 0.05; ***, *P* < 0.001; ****, *P* < 0.0001 (by one-way ANOVA with Dunnett’s posttest for multiple comparisons to the control group [uninfected mice]). *Zbp1* data (C) did not pass a normality test and were not subjected to further statistical analysis. (F) LCN-2 in stool samples measured by ELISA. Each point (at individual time points) represents an individual mouse; samples at each day postinfection are from the same mice, collected over time. Data are from 3 biological repeats. *, *P* < 0.05; ***, *P* < 0.001; ****, *P* < 0.0001 (by RM one-way ANOVA with Dunnett’s posttest for multiple comparisons to the control group [0 DPI]). (I) z scores of ferritin (FTL1/FTH1) and MT2 protein abundances across the entire C. rodentium infection time course (4 to 48 DPI). Means ± SEM are shown.

We have shown previously that C. rodentium induces a strong cIEC inflammatory response during the expansion phase and at the entry to the steady-state infection phase. Analyzing the proteomic responses during the later infection phases revealed that immune processes continued to be enriched (Fig. 1D), and many innate immunity proteins were identified in the 100 most highly changed proteins at 10 to 20 DPI (Fig. S3A). Immune-related proteins, including NOS2; the antimicrobial peptides REG3β, REG3γ, and CAMP; as well the cytosolic nucleic acid sensors ZBP1 (DAI), cGAS, TMEM173 (STING), IFIH1 (MDA5), DDX58 (RIG-I), and AIM2, were significantly upregulated during the late steady-state and clearance phases (Fig. 3A and B), with *Zbp1* and *Tmem173* being transcriptionally upregulated at 10 to 20 DPI and 10 to 13 DPI, respectively (Fig. 3C and D), while *Aim2* expression was unchanged at the transcriptional level (Fig. 3E). Moreover, the levels of several factors involved in nutritional immunity, including DMBT1, S100A8/A9, lipocalin-2 (LCN-2), lactotransferrin (LTF), haptoglobin (HP), hemopexin (HPX) and the transferrin receptor (TFRC), were strongly elevated during the clearance phase of infection, peaking at 13 DPI before recovering by 48 DPI (Fig. 3A). In agreement with these results, enzyme-linked immunosorbent assay (ELISA) measurements of LCN-2 excreted into the stool mirrored this pattern (Fig. 3F). Conversely, metallothionein-2 (MT2), a secreted protein that mediates zinc uptake across the apical membrane, and ferritin (FTL1/FTH1), which stores free cellular iron, were both transcriptionally downregulated and found in lower abundances during the late infection phases, failing to recover by 48 DPI (Fig. 3A, G to I), suggesting that changes to cIEC metal ion homeostasis have not recovered by 48 DPI. Overall, these results show that cIEC innate immune responses to C. rodentium infection peak at around 13 DPI, coinciding with the clearance of mucosa-associated C. rodentium, and are largely resolved by 48 DPI, although some remnants of infection-associated immune and nutritional responses remain.

10.1128/mBio.03233-21.3 FIG S3 The top changed proteins during the clearance (10 to 20 DPI) and recovery (48 DPI) phases (related to Fig. 2 and 3). (A) Average Log_2_FC values of the 100 “top changed” proteins during the clearance phase time points (10 to 20 DPI). Average Log_2_FC values of these proteins at 48 DPI are shown for reference. (B) Average Log_2_FC values of all proteins considered significantly changed at 48 DPI versus the UI controls (FDR of <0.05 by Tukey’s posttest). Log_2_FC values of these proteins at 10 to 20 DPI are shown for reference. Download FIG S3, PDF file, 0.2 MB. Copyright © 2022 Mullineaux-Sanders et al. 2022 Mullineaux-Sanders et al. https://creativecommons.org/licenses/by/4.0/ This content is distributed under the terms of the Creative Commons Attribution 4.0 International license.

### The recovery phase is characterized by activation of antigen presentation processes.

We next focused on processes and proteins that are altered during infection and remain significantly changed at 48 DPI, 4 weeks after the infection has effectively been eliminated, thus representing long-term, infection-induced perturbations in the colonic epithelium (Fig. S3B). The most striking change in protein expression during and after the clearance phase of C. rodentium infection was the upregulation of proteins involved in antigen processing and presentation, which was among the most highly enriched processes (Fig. 1D). Indeed, 27 of the 33 identified proteins assigned to the KEGG process “antigen processing and presentation” were considered significantly changed, and many major histocompatibility complex class I (MHCI) and MHCII molecules, and the intracellular proteins required for peptide processing, were among the most highly changed proteins (Fig. S3A and B).

**FIG 4:**
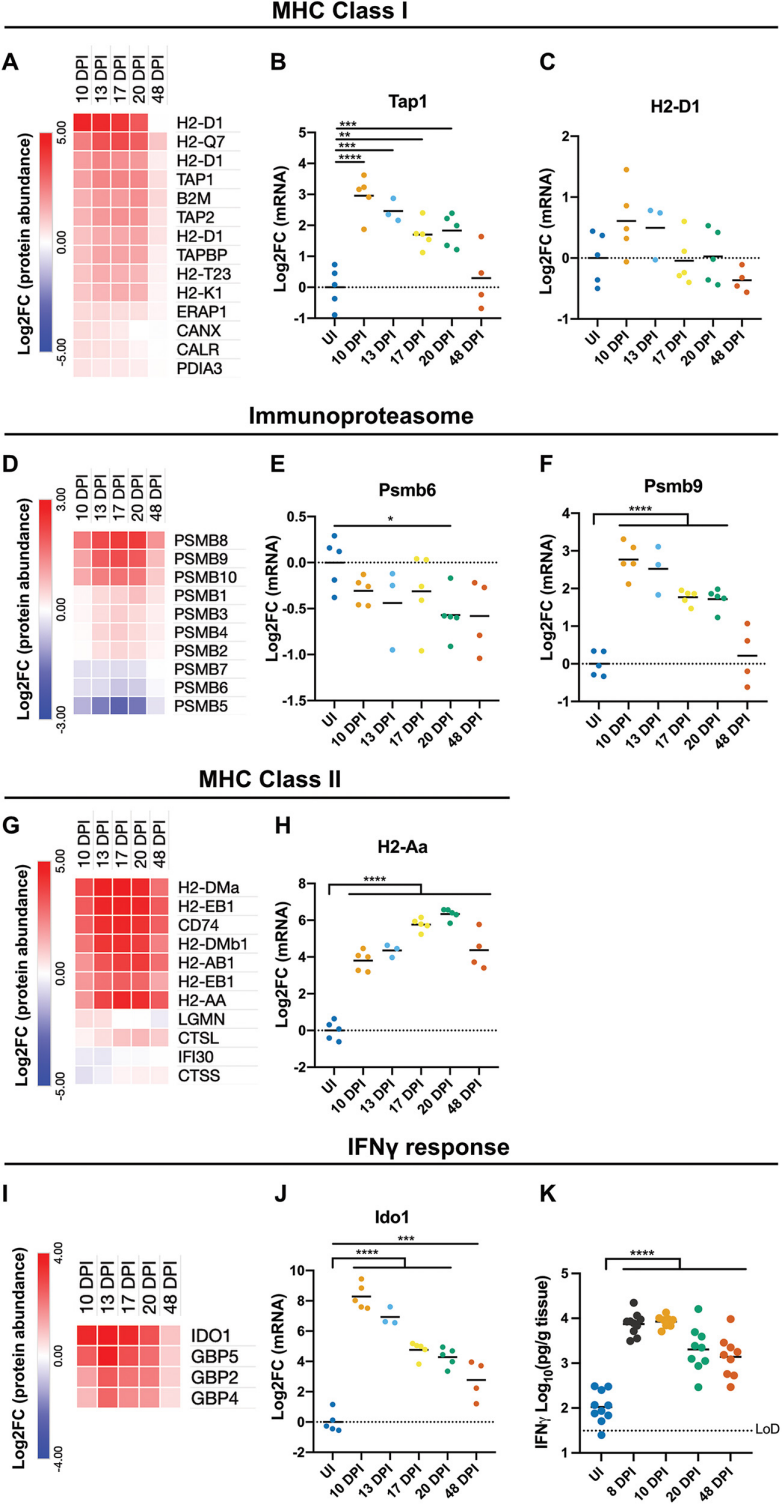
cIECs express MHCII proteins 4 weeks after C. rodentium clearance. (A, D, G, and I) Average Log_2_FC values of selected proteins required for MHCI antigen processing and presentation (A), the constitutive proteasome and immunoproteasome (D), MHCII antigen processing and presentation (G), and selected IFN-γ-regulated proteins (I). All proteins shown were significantly changed (FDR of <0.05 by one-way ANOVA). (B, C, E, F, H, and J) qRT-PCR of the indicated mRNAs isolated from cIECs. *, *P* < 0.05; **, *P* < 0.01; ***, *P* < 0.001; ****, *P* < 0.0001 (by one-way ANOVA with Dunnett’s posttest for multiple comparisons to the control group [uninfected mice]). (K) IFN-γ levels in distal colonic explants at the indicated days after infection with C. rodentium. ****, *P* < 0.0001 (by one-way ANOVA with Dunnett’s posttest for multiple comparisons to the control group [uninfected mice]). Each point represents an individual mouse. Data are from two biological repeats. LoD, limit of detection.

Proteins involved in MHCI antigen processing and presentation, which typically present endogenous antigens, were strongly upregulated at 10 to 20 DPI (Fig. 4A). Six out of nine identified MHCI alpha chains and β2-microglobin were significantly changed, with peak log_2_ fold change (Log_2_FC) values of between 1.6 and 4.5. Likewise, calnexin (the heavy chain chaperone) was also significantly upregulated between 10 and 17 DPI. Additionally, other members of the peptide-loading complex, TAP1, TAP2, tapasin (TAPBP), calreticulin (CALR), ERP57 (PDIA3), and ERAP1 (required for peptide trimming), were all significantly upregulated during the clearance phase of infection (Fig. 4A); *Tap1* was significantly transcriptionally upregulated at 10 to 20 DPI (Fig. 4B), while the MHCI protein H2-D1 showed a modest transcriptional increase at 10 to 13 DPI (Fig. 4C). The elevation of MHCI antigen processing and presentation proteins was resolved between 20 and 48 DPI, with no proteins being significantly upregulated at 48 DPI compared to the UI controls (FDR of <0.05 by Tukey’s posttest) (Fig. 4A).

Intracellular peptides are degraded by specialized subunits of the proteasome for presentation by MHCI molecules; consistently, we observe a robust switch from the constitutive proteasome to the immunoproteasome during the clearance phase of infection. The core subunits PSMB1 to -4 were modestly, but significantly, elevated (peaking at 17 to 20 DPI), while the constitutive proteasome catalytic subunits PSMB5, PSMB6, and PSMB7 were significantly decreased in abundance (Fig. 4D); *Psmb6*, as a representative gene, was also transcriptionally downregulated (Fig. 4E). Concomitantly, we observe a strong enrichment of the immunoproteasome catalytic subunits PSMB8, PSMB9, and PSMB10 (Fig. 4D); *Psmb9* was significantly transcriptionally upregulated from 10 to 20 DPI (Fig. 4F). Furthermore, PSMB8, PSMB9, and PSMB10 remained elevated at 48 DPI compared to the UI controls.

All identified MHCII proteins, which typically present exogenous antigens, as well as the invariant chain (CD74), were strongly elevated throughout the clearance phase of infection, with a peak Log_2_FC of between 3.1 and 4.9 (Fig. 4G). Interestingly, in contrast to the MHCI antigen presentation pathway, MHCII presentation proteins remained strongly elevated after C. rodentium clearance, with Log_2_FC values of 2.7 to 3.2 at 48 DPI (Fig. 4G). Indeed, of the proteins considered significantly changed at 48 DPI compared to the UI controls (FDR of <0.05 by Tukey’s posttest) (Fig. S3B), 5 of the top 10 most highly elevated proteins are involved in MHCII presentation. The strong transcriptional upregulation, persisting until 48 DPI, of *H2-Aa* was validated by quantitative reverse transcription PCR (qRT-PCR) (Fig. 4H). These data demonstrate that strong local MHCII expression persists throughout the host recovery phase.

Many of the proteins found to remain significantly altered in abundance at 48 DPI, including MHCII proteins, PSMB8, PSMB10, and WFDC2, are IFN-γ regulated; moreover, the IFN-γ-regulated proteins, IDO1 and selected guanylate-binding proteins (GBPs), showed increased protein abundances at 48 DPI (Fig. 4I), with *Ido1* remaining significantly transcriptionally upregulated at 48 DPI (Fig. 4J). Importantly, measurement of IFN-γ in whole colonic tissue explants showed a significant increase in IFN-γ at 8 DPI, which remained elevated until 48 DPI (Fig. 4K), while IL-22 levels returned to baseline levels (Fig. S4A), suggesting that IFN-γ underpins the long-term changes to cIECs following C. rodentium infection.

**FIG 5:**
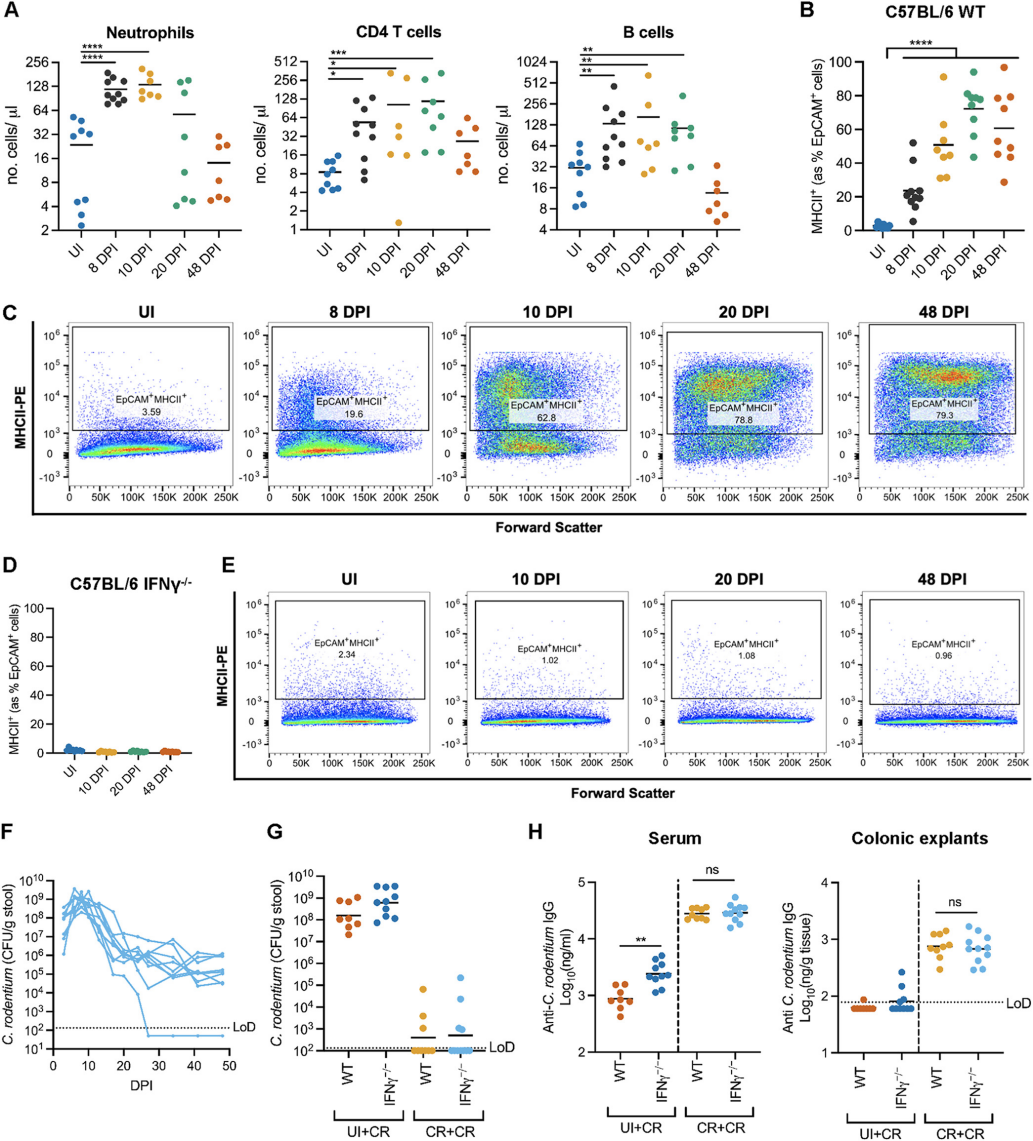
IFN-γ and cIEC MHCII expression are not required for protective immunity against C. rodentium. (A) Quantification of the indicated immune cell types in colonic tissue during C. rodentium infection in WT mice. Each point represents an individual mouse, and data are from two biological repeats. *, *P* < 0.05; **, *P* < 0.01; ***, *P* < 0.001; ****, *P* < 0.0001 (by one-way ANOVA with Dunnett’s posttest for multiple comparisons to the control group [uninfected mice]). (B and C) Quantification (B) and representative flow cytometry plots (C) of EpCAM^+^ cIECs expressing MHCII (anti-IA/IE staining) in uninfected or C. rodentium-infected WT mice. Each point represents an individual mouse. Data are from two biological repeats. ****, *P* < 0.0001 (by one-way ANOVA with Dunnett’s posttest for multiple comparisons to the control group [uninfected mice]). (D and E) Quantification (D) and representative flow cytometry plots (E) of EpCAM^+^ cIECs expressing MHCII (anti-IA/IE staining) in uninfected or C. rodentium-infected *Ifn*γ^−/−^ mice. Each point represents an individual mouse. Data are from two biological repeats. (F) C. rodentium colonization of *Ifn*γ^−/−^ mice. Each line represents an individual mouse. Data are from two biological repeats. (G) C. rodentium colonization at 10 DPI following reinfection of WT and *Ifn*γ^−/−^ mice initially mock infected (UI+CR) or infected with C. rodentium (CR+CR). Each point represents an individual mouse. Data are from two biological repeats. (H) ELISA measurement of anti-C. rodentium IgG in the serum (left) and colonic explants (right) of the mice shown in panel G, 10 days after the second infection. ns, not significant (*P* > 0.05); **, *P* < 0.01 (by unpaired two-tailed Student’s *t* test between the indicated groups). UI+CR colonic explant data (right) were not statistically analyzed due to the high number of samples falling below the limit of detection (LoD).

10.1128/mBio.03233-21.4 FIG S4 The role of IFN-γ in protective immunity (related to Fig. 4 and 5). (A) IL-22 levels measured in distal colonic explants at the indicated days after infection with C. rodentium. Each point represents an individual mouse. Data are from two biological repeats. Data were not statistically analyzed due to the high number of samples falling below the limit of detection (LoD). (B) Quantification of the indicated immune cell types in colonic tissue during C. rodentium infection in WT mice. Each point represents an individual mouse, and data are from two biological repeats. **, *P* < 0.01 (by one-way ANOVA with Dunnett’s posttest for multiple comparisons to the control group [uninfected mice]). (C) Schematic of the rechallenge experimental design (top) and C. rodentium colonization of WT (middle) (in orange) and *Ifn*γ^−/−^ (bottom) (in blue) mice. Black triangles denote the times of C. rodentium inoculations. Mice were mock infected with PBS at 0 DPI and infected with Kan^r^ C. rodentium (ICC180) at 52 DPI (UI+CR) or infected with Nal^r^ C. rodentium (ICC169) at 0 DPI and reinfected with Kan^r^ C. rodentium ICC180 at 52 DPI (CR+CR). All mice were given oral ciprofloxacin treatments at 42, 43, and 45 DPI (treatment period denoted by vertical dotted lines in the line plots). Each line represents an individual mouse, and data are from two biological repeats. o.g., oral gavage. (D) Quantification of the indicated immune cell types in colonic tissue of UI+CR and CR+CR WT and *Ifn*γ^−/−^ mice 10 days after reinfection with ICC180. ns, not significant (*P* > 0.05) (by an unpaired *t* test between the indicated groups). Each point represents an individual mouse, and data are from two biological repeats. Download FIG S4, PDF file, 0.4 MB. Copyright © 2022 Mullineaux-Sanders et al. 2022 Mullineaux-Sanders et al. https://creativecommons.org/licenses/by/4.0/ This content is distributed under the terms of the Creative Commons Attribution 4.0 International license.

### The role of cIEC MHCII presentation in protective immunity against reinfection.

We next investigated the role of IFN-γ and the expression of MHCII molecules on cIECs in recovery from C. rodentium infection and protection against reinfection. IFN-γ is secreted in the colon by infiltrating immune cells, and in agreement, analysis of colonic tissue during C. rodentium infection showed a general increase in myeloid and lymphocytic cell lineages from 8 to 20 DPI (Fig. 4K and Fig. 5A; Fig. S4B), with the exception of conventional dendritic cell (cDC) numbers (CD11b^+^ DCs), which showed minimal changes over the infection course (Fig. S4B). As expected, most cell types, including B cells and neutrophils, returned to preinfection levels during the host recovery phase. Conversely, CD4^+^ T cells, which are reported to secrete IFN-γ during and after C. rodentium infection (31), remained elevated above mock-infected levels, albeit not significantly, at 48 DPI (Fig. 5A).

To characterize the progression of MHCII expression by cIECs during C. rodentium infection at the single-cell level, we analyzed cIECs using flow cytometry. This revealed that around 2% of UI cIECs (EpCAM^+^ cells) expressed MHCII, which increased gradually to 70% by 20 DPI and remained similarly elevated (at around 60% MHCII^+^ cIECs) at 48 DPI (Fig. 5B and C). Conversely, there was no increase in the cIEC expression of MHCII in IFN-γ KO (*Ifn*γ*^−/−^*) mice, demonstrating that IFN-γ is necessary for the induction of MHCII expression on cIECs during C. rodentium infection (Fig. 5D and E). *Ifn*γ*^−/−^* mice were unable to fully clear the initial C. rodentium infection and persistently harbored C. rodentium at around 10^6^ CFU/g stool from 20 to 48 DPI (Fig. 5F), suggesting that IFN-γ plays either a direct or an indirect role in full C. rodentium clearance.

Canonical antigen-presenting cells (APCs) typically present exogenous antigens via MHCII to CD4^+^ T cells, which subsequently induce antibody (including IgG) production by plasma B cells and the generation of memory B cells (32, 33). To test whether prolonged secretion of IFN-γ, and the resulting expression of MHCII molecules on cIECs, is required for protection against reinfection, WT and *Ifn*γ*^−/−^* mice were infected with C. rodentium or mock infected with phosphate-buffered saline (PBS) as a control. All mice received ciprofloxacin treatment at 42 to 45 DPI, to clear persistently colonizing C. rodentium in *Ifn*γ*^−/−^* mice, and then rechallenged with C. rodentium at 52 DPI. As expected, control WT and *Ifn*γ*^−/−^* mice that were initially mock infected developed a normal C. rodentium infection after the second challenge (UI+CR) (Fig. 5G; Fig. S4C). Conversely, rechallenged WT and *Ifn*γ*^−/−^* mice (CR+CR) displayed protective immunity against C. rodentium reinfection (Fig. 5G; Fig. S4C). This was correlated with comparable immune cell profiles (Fig. S4D) and levels of anti-C. rodentium serum and colonic IgG in both WT and *Ifn*γ*^−/−^* mice (Fig. 5H). Together, these data demonstrate that the long-term presence of IFN-γ following infection and the ensuing cIEC MHCII antigen presentation are not required for protection against reinfection with C. rodentium.

## DISCUSSION

Studies of mucosal barrier perturbations have mainly focused on the acute phase of the disease at a single time point, but little is currently known about the dynamics of the resolution and regeneration phases or the molecular processes that underpin the recovery process and return to homeostasis in epithelial cells. We addressed this knowledge gap by characterizing the temporal cellular responses of cIECs using quantitative proteomics across the late steady-state (10 DPI), clearance (13 to 20 DPI), and subsequent host recovery (20 to 48 DPI) phases using C. rodentium infection as a model. We found that the cIEC innate inflammatory responses to C. rodentium and infection-induced changes to metabolism and bioenergetics are largely resolved following pathogen clearance, at between 20 and 48 DPI, which we define as the “host recovery” phase of infection. A residual elevation in the level of the glucose transporter SLC5A9 remained at 48 DPI, suggesting that there may be a lag between the return of the proteins to preinfection levels and the full restoration of homeostatic bioenergetic pathways.

Surprisingly, we find that while morphologically (i.e., CCH and PCNA staining) the mucosa has recovered 4 weeks following C. rodentium clearance, on the molecular and cellular levels the cIECs have not fully reset to the preinfection homeostatic state. The panendocrine cell markers CHGA and CHGB sharply decrease from 8 to 20 DPI and remain decreased at 48 DPI. In healthy tissue, enteroendocrine cells are located throughout the colonic crypt and secrete hormones; they consist of several specialist cells types, of which enterochromaffin (EC) cells, which secrete serotonin (5-hydroxytryptamine [5-HT]), are the most abundant (34, 35). EC cells play a functional role in C. rodentium pathogenesis as serotonin has been shown to repress C. rodentium virulence *in vivo* (36), and we have previously shown CHGA/CHGB to be weakly upregulated during early infection (20), when C. rodentium adapts to the host environment and turns on virulence gene expression. Levels of WFDC2, a goblet cell-secreted antibacterial protein that is negatively regulated by IFN-γ (30), also remained significantly decreased at 48 DPI. WFDC2 was found to be downregulated in human ulcerative colitis (UC) colonic samples compared to healthy patients, and *Wfdc2*^+/−^ mice show abnormalities in colonic epithelial intercellular junctions (30). Although goblet cell numbers recovered by 20 DPI, WFDC2 remained at a reduced abundance at 48 DPI. Overall, these data suggest that the integrity and cellular composition of the colonic epithelial cell barrier may not be fully restored.

Interestingly, we observed an increased abundance of proteins involved in cytosolic nucleic acid sensing, mainly characterized as antiviral sensors, during the clearance phase of C. rodentium infection (13 to 20 DPI). While the upregulation of these sensors may be a bystander effect of the increased levels of IFN-γ, they could also play an active role in the clearance of the pathogen and the onset of tissue recovery via the induction of type I IFN responses, which have been linked to the maintenance of gut homeostasis and tissue repair (37) and inflammatory cell death pathways. For example, necroptosis likely plays a role in pathogen clearance as C. rodentium lacking the T3SS effector EspL, which inhibits necroptotic cell death pathways, is cleared quicker from the colon (38). The nature of the ligands activating the sensors needs further investigation, but it is likely that both endogenous mitochondrial DNA (39), liberated in response to C. rodentium-induced mitochondrial damage, and exogenous nucleic acids from the microbiota (40) can activate these sensors. Additionally, EHEC nucleic acids have been shown to lead to cytosolic NLRP3 inflammasome activation, opening the possibility of C. rodentium DNA/RNA being the ligand itself (41).

We find that the host recovery phase of C. rodentium infection is characterized by a strong IFN-γ response, which persists for 4 weeks after the pathogen has been cleared. The most striking IFN-γ-induced change to the cIECs was the continued expression of MHCII antigen presentation proteins at 48 DPI. While MHCII molecules are constitutively expressed in small intestinal IECs (siIECs), cIECs have been shown to express MHCII in response to IFN-γ (42), and we find that *Ifn*γ*^−/−^* mice do not show an increase in MHCII^+^ cIECs during or after C. rodentium infection, confirming that IFN-γ is essential in this context. The role of cIEC MHCII expression during and after infection is not well understood. The induction of protective immunity against C. rodentium is dependent on the induction of local (gut) immune responses, while mice with high levels of systemic (but not local) C. rodentium-specific IgG alone remain susceptible to reinfection (43), raising the possibility that cIECs may play a role as nonconventional antigen-presenting cells, promoting immune memory responses in the colon. While we cannot fully rule out a contributing role, we find that *Ifn*γ*^−/−^* and WT mice are equally protected against rechallenge, demonstrating that IFN-γ-dependent cIEC MHCII expression is not necessary for IgG-mediated protective immunity against C. rodentium.

The benefit to the host of continued IFN-γ expression in the colon for weeks after pathogen clearance is currently unknown; however, it is possible that the resulting cIEC MHCII may have a role in mucosal regeneration or a noncanonical immunomodulatory effect. Small intestinal epithelial stem cells act as nonprofessional antigen-presenting cells via MHCII, which assist in epithelial regeneration following infection (44). Although the high percentage (60 to 70%) of cIECs that express MHCII during and after C. rodentium infection suggests that this is not confined to colonic stem cells, it is plausible that cIEC MHCII may also play a similar role in colonic epithelium renewal. Additionally, a possible role for cIEC MHCII in favoring an immunosuppressive response during intestinal inflammation via the activation of regulatory T cells has been suggested, as shown by a study showing that mice lacking MHCII in nonhematopoietic cells developed more severe T cell-mediated colitis (45). Taken together with the fact that *Ifn*γ*^−/−^* mice develop increased mucosal damage 14 days after infection with C. rodentium (25), it is reasonable to consider that cIECs could play a similar role during and after C. rodentium infection. Accordingly, we rule out a role for cIEC MHCII in the generation of B cell-dependent protective immunity, suggesting that the focus should now be shifted to understanding what role they play postinfection in the recovery of tissue homeostasis.

## MATERIALS AND METHODS

### Bacterial growth and maintenance.

C. rodentium strains ICC169 (46, 47) and ICC180 (19) were used in this study. C. rodentium was grown in lysogeny broth (LB; VWR) at 37°C with shaking at 200 rpm unless otherwise specified. For CFU quantification, C. rodentium was grown on LB solidified with 1.5% agar (LBA; VWR). Where necessary, the following antibiotics were added: nalidixic acid at 50 μg/μL and kanamycin at 50 μg/μL.

### Mouse experiments.

Mouse experiments were performed in accordance with the Animals Scientific Procedures Act of 1986 and were approved by the local Ethical Review Committee according to UK Home Office guidelines. Specific-pathogen-free female C57BL/6 mice (18 to 20 g) were purchased from Charles River, London, United Kingdom, and housed in cages of five. For each experiment, five mice were randomly assigned to experimental groups. Investigators were not blind to the allocation. C57BL/6J IFN-γ knockout mice (B6.129S7-*Ifng*tm^1Ts/J^; Jackson Laboratories) (referred to as *Ifn*γ^−/−^ throughout) and C57BL/6J WT control mice (Jackson Laboratories) were bred at Imperial College London and housed in cages of one to four. Male and female mice (8 to 12 weeks old) were allocated to experimental groups to ensure even distributions of age and sex. Investigators were not blind to the allocation. All mice used in this study were housed in individually HEPA-filtered cages with bedding and nesting and given free access to food and water. Mice that lost 20% of their initial weight or became moribund during the experimental period were humanely culled and excluded from all analyses (see Table S3 in the supplemental material). Where mice were purchased from a supplier, we used female mice only to avoid the logistical issues and stress to animals associated with male in-cage fighting. Where mice were locally bred, both sexes were used due to the limited availability of single-sex animals.

10.1128/mBio.03233-21.8 TABLE S3 Number of mice excluded from all analyses in each experiment and the reasons behind the exclusion. Download Table S3, DOCX file, 0.02 MB. Copyright © 2022 Mullineaux-Sanders et al. 2022 Mullineaux-Sanders et al. https://creativecommons.org/licenses/by/4.0/ This content is distributed under the terms of the Creative Commons Attribution 4.0 International license.

### C. rodentium infection.

Mice were orally gavaged with approximately 1 × 10^9^ CFU C. rodentium (strain ICC169 unless otherwise specified) in 200 μL sterile PBS as previously described (21). Mock-infected (“uninfected”) mice received 200 μL sterile PBS. The inoculum CFU was retrospectively confirmed by CFU quantification as previously described (21). C. rodentium stool shedding was determined by collecting stool samples at the specified days postinfection, homogenizing the samples in PBS, and plating serial dilutions onto LBA containing selective antibiotics. For rechallenge experiments, mice in all groups were orally gavaged with 2 mg ciprofloxacin at 42, 43, and 45 DPI and rechallenged by oral gavage with approximately 1 × 10^9^ CFU C. rodentium (strain ICC180) at 52 DPI. Any mouse that did not reach a C. rodentium colonization threshold of 1 × 10^8^ CFU/g stool at 6 DPI (first C. rodentium infection) was excluded from all analyses (Table S3); no C. rodentium colonization threshold was implemented for rechallenge with ICC180. After the exclusion of cIEC samples based on these criteria, all proteomics samples consisted of cIECs pooled from at least 4 mice per group in each biological repeat.

### Histopathological analysis of colonic tissue.

Formalin-fixed 0.5-cm distal colon samples were processed, paraffin embedded, and sectioned at 5 μm. Formalin-fixed, paraffin-embedded (FFPE) sections were then stained with either hematoxylin and eosin (H&E) or alcian blue and periodic acid-Schiff (AB/PAS) stain using standard techniques. Images were acquired using a Zeiss AxioVision Z3 microscope with a 20× lens objective and processed using Zen 2.3 (Blue version; Carl Zeiss MicroImaging GmbH, Germany). CCH and goblet cell density measurements were obtained from at least 10 well-oriented crypts per mouse. Colonic sections from which fewer than 10 well-orientated crypts were observed were excluded from all analyses. CCH measurements were performed on H&E-stained sections. The goblet cell density was measured on AB/PAS-stained sections. The goblet cell density (goblet cells/100 μm crypt) for each mouse was calculated by dividing the total number of AB/PAS-positive cells counted exclusively from the crypts by the total crypt length of the crypts from which they were counted.

### Immunofluorescence staining of colonic tissue.

FFPE sections were processed and stained as previously described (20). Images were acquired and processed as described above for histopathological analysis. The following primary antibodies were used: rabbit polyclonal anti‐C. rodentium antibody (1:50) (Statens Serum Institute, Copenhagen, Denmark), mouse anti-PCNA antibody (1:500) (catalog number ab29; Abcam), and rabbit anti-CHGB antibody (1:50) (catalog number 14968-1-AP; Proteintech). The following secondary antibodies were used: Alexa Fluor 555 anti-rabbit and rhodamine Red-X donkey anti-mouse (both 1:100) (Jackson Immunoresearch). DNA was stained with Hoechst 33342 or 4′,6-diamidino-2-phenylindole (DAPI) (1:1,000). PCNA measurements were obtained from at least 10 well-oriented crypts per mouse. Colonic sections from which fewer than 10 well-orientated crypts were observed were excluded from all analyses. The endocrine cell density (cells per square millimeter of crypt area) for each mouse was calculated by dividing the total number of CHGB-positive cells within the crypt area by the total crypt area. The total crypt area was calculated by tracing around the edges of colonic crypts and the lumen of the gut using the Contour(polygon) tool in Zen 2.3 (Blue version; Carl Zeiss MicroImaging GmbH, Germany).

### cIEC purification.

cIECs were isolated from 3.5-cm distal colonic tissue samples (following the removal of the most distal 1-cm tissue for histological analysis), as previously described (20). Briefly, the 3.5-cm colonic tissue sample was opened longitudinally and briefly washed in 1× Hanks’ balanced salt solution (HBSS) without Mg and Ca. The tissue sample was incubated at 37°C with shaking for 45 min in enterocyte dissociation buffer (1× HBSS without Mg and Ca, containing 10 mM HEPES, 1 mM EDTA, and 5 μL/mL 2-β-mercaptoethanol). The remaining tissue was removed, and lifted enterocytes were subsequently collected by centrifugation (2,000 × *g* for 10 min), followed by three PBS washes at 4°C. cIEC pellets were stored at −80°C.

### Sample preparation and TMT labeling.

cIEC pellets isolated from 4 to 5 mice per group (time point) per biological replicate were individually solubilized using probe sonication in lysis buffer (100 mM triethylammonium bicarbonate [TEAB], 1% sodium deoxycholate [SDC], 10% isopropanol, 50 mM NaCl) supplemented with Halt protease and phosphatase inhibitor cocktail (Thermo Scientific), boiled for 5 min at 90°C, and sonicated once more. The protein concentration was determined with a Coomassie Plus assay (Thermo Scientific) according to the manufacturer’s protocol. Equal amounts of protein from each mouse from each group were combined. A total of 100 μg of protein per group was reduced with 5 mM Tris-2-carboxyethyl phosphine (TCEP) for 1 h, followed by alkylation with 10 mM iodoacetamide (IAA) for 30 min, and then digested by adding trypsin (Pierce) at a final concentration of 75 ng/μL to each sample and incubating the samples for 18 h at room temperature. Peptides were labeled with tandem mass tag (TMT) multiplex reagent (Thermo Scientific) for 1 h before quenching with a final volume of 5% hydroxylamine for 15 min (Sigma). TMT-labeled peptides were combined at equal amounts, and SDC was precipitated with formic acid (FA) at a final concentration of 2% (vol/vol) and centrifugation for 5 min at 10,000 rpm. The supernatant containing TMT-labeled peptides was dried with a centrifugal vacuum concentrator.

### High-pH reversed-phase peptide fractionation.

TMT-labeled peptides were reconstituted in 0.1% ammonium hydroxide and fractionated using a high-pH reversed-phase (RP) Waters XBridge C_18_ column (2.1 by 150 mm, 3.5 μm) on a Dionex Ultimate 3000 high-performance liquid chromatography (HPLC) system. Mobile phase A was 0.1% ammonium hydroxide, and mobile phase B was 100% acetonitrile and 0.1% ammonium hydroxide. A gradient elution at 200 μL/min was used to separate the peptides in the following steps: isocratic for 5 min at 5% phase B, gradient for 40 min to 35% phase B, gradient to 80% phase B in 5 min, isocratic for 5 min, and reequilibration to 5% phase B. A total of 65 fractions were collected every 42 s into a 96-well plate, dried, and concatenated into 28 fractions upon reconstitution in 0.1% formic acid.

### Liquid chromatography-mass spectrometry analysis.

All data were acquired using an Orbitrap Fusion mass spectrometer (Thermo Scientific) coupled to a Dionex Ultimate 3000 system. The mobile phases were 0.1% FA (phase A) and 80% acetonitrile in 0.1% FA (phase B). Samples were loaded onto a C_18_ trapping column (Acclaim PepMap 100, 100 μm by 2 cm, 5 μm, 100 Å) at a 10-μL/min flow rate, followed by low-pH gradient elution on a nanocapillary reversed-phase column (Acclaim PepMap C_18_, 75 μm by 50 cm, 2 μm, 100 Å) at 45°C. A flow rate of 300 nL/min was applied to separate the peptides over a 90-min gradient from 5% to 38% phase B followed by 10 min up to 95% phase B, isocratic for 5 min at 95% phase B, reequilibration to 5% phase B in 5 min, and isocratic for 10 min at 5% phase B. The instrument method included Orbitrap MS1 scans (automatic gain control [AGC] of 4 × 10^5^, maximum injection time of 50 ms, and resolution of 120,000), ion trap MS2 scans (top-speed mode [3 s], collision-induced dissociation [CID] energy of 35% with a quadrupole isolation width of 0.7 Th, AGC target of 1 × 10^4^, and maximum injection time of 50 ms), and MS3 scans of the top seven most abundant CID fragments isolated with synchronous precursor selection (resolution of 50,000, mass range of *m/z* 100 to 500, higher-dissociation collision energy of 65%, AGC target of 1 × 10^5^, and injection time of 105 ms).

### Protein identification and TMT-based quantification.

Proteome Discoverer 2.3 (Thermo Scientific) using the SEQUEST-HT algorithm was employed to search MS/MS spectra against UniProt annotated reference proteomes of Mus musculus and Citrobacter rodentium. Searches were performed with a 20-ppm precursor mass tolerance, a 0.5-Da fragment ion mass tolerance, and trypsin digestion with up to two missed cleavages. The static modifications were specified as carbamidomethylation of Cys residues (+57.02146) and TMT modification of peptide N-terminal and Lys residues (+229.16293), while dynamic modifications were specified as oxidation of Met (+15.99491) and deamidation of Asp and Glu residues. The confidence of peptide discovery was estimated at a 1% FDR with the percolator node based on the *q* value and decoy database search. The reporter ion quantifier node included a TMT 10-plex quantification method with an integration window tolerance of 15 ppm and an integration method based on the most confident centroid peak at the MS3 level. Protein groups with only unique peptides were used for quantification.

### LCN-2 ELISA.

Stool samples were homogenized in PBS with 0.1% Tween 20 using a vortex machine for 15 min. Samples were centrifuged at 16,000 rpm for 10 min, and the supernatant was extracted and stored at −80°C. The LCN-2 concentration was determined using a DuoSet mouse lipocalin-2 ELISA (R&D Systems), according to the manufacturer’s instructions.

### IFN-γ and IL-22 ELISAs.

A 0.5-cm distal colonic tissue sample without feces was weighed and washed for 2 h in RPMI 1640 medium with glutamine (Sigma) with 100 μg/mL of streptomycin and 100 μg/mL of penicillin to remove residual cytokines present on the tissue surface. Explants were then rinsed and cultured in complete RPMI medium (RPMI 1640 medium with glutamine [Sigma] supplemented with 10% heat-inactivated fetal bovine serum [FBS] [Gibco], 1 mM sodium pyruvate, 100 μg/mL penicillin, 100 μg/mL streptomycin, and 10 mM HEPES [all from Sigma]) for 24 h at 37°C with 5% CO_2_. The supernatant was extracted, centrifuged for 10 min at 3,000 × *g* to remove cellular debris, and stored at −80°C. IFN-γ levels were determined using an IFN-γ Femto-HS high-sensitivity mouse uncoated ELISA kit (Thermo Fisher Scientific), and IL-22 levels were determined using an IL-22 mouse uncoated ELISA kit (Thermo Fisher Scientific), in both cases according to the manufacturer’s instructions.

### Anti-C. rodentium IgG ELISA.

For measurement of colonic IgG, 0.5-cm distal colon explants were prepared as described above for the IFN-γ ELISA. For serum measurements, blood was extracted from mice after cervical dislocation and allowed to coagulate at room temperature for at least 3 h. Blood was then centrifuged at 2,000 rpm for 15 min to pellet the blood clots, and the supernatant (serum) was collected and stored at −80°C. To prepare heat-killed C. rodentium, ICC169 was grown to saturation in LB at 37°C at 200 rpm, diluted 1:50 in low-glucose Dulbecco’s modified Eagle’s medium (DMEM) (Sigma), and grown overnight at 37°C with 5% CO_2_ to stimulate T3SS expression. The culture was then normalized to an optical density (at 600 nm) of 0.4, heat inactivated in the presence of EDTA-free Pierce protease inhibitor cocktail (Thermo Fisher Scientific) at 60°C for 1 h, and finally diluted 20-fold in PBS. Immuno 96-well plates (Thermo Fisher Scientific) were incubated with 50 μL/well heat-killed C. rodentium for the samples or 100 μL/well goat anti-mouse IgG antibody for the standards (2 μg/μL) (Poly4053; BioLegend) overnight at 4°C. Plates were washed three times with PBS plus 0.1% Tween 20, blocked for 1 h at room temperature with PBS plus 0.1% Tween 20 and 5% bovine serum albumin (BSA), and incubated with samples or serial dilutions of purified mouse serum IgG as the standard (Merck) overnight at 4°C. Plates were washed as described above and incubated with horseradish peroxidase (HRP)-conjugated goat anti-mouse IgG antibody (Jackson Immunoresearch) (1:2,000 dilution) for 2 h at room temperature. Plates were washed as described above and developed by the addition of 100 μL 3,3′,5,5′-tetramethylbenzidine (TMB; Thermo Fisher Scientific) for 5 to 10 min before quenching with 2 N H_3_PO_4_. The absorbances at 450 and 540 nm were measured in a plate reader (FLUOstar Omega; BMG Labtech), and anti-C. rodentium IgG was quantified in samples by comparison to a mouse IgG standard curve.

### Quantitative reverse transcription-PCR (qRT-PCR).

RNA was isolated from extracted cIECs using the RNeasy minikit (Qiagen) according to the manufacturer’s instructions. RNA was treated with RQ1 RNase-free DNase (Promega) for 30 min, and subsequently, cDNA was synthesized using a Moloney murine leukemia virus reverse transcription kit (Promega). Amplification from cDNA was performed using Power SYBR green PCR master mix (Thermo Fisher Scientific). Assay reactions were performed in a 20-μL volume with 2× Power SYBR green PCR master mix, efficiency-optimized primers to a final concentration of 0.05 μM each, and 1 to 10 ng total cDNA. All reactions were carried out in technical duplicate. The ΔΔ*C_T_* method of quantification was performed to give Log_2_FC values relative to the expression of the averaged baseline measurement (48). Expression was normalized to the expression of the housekeeping gene *Gapdh*. Primer pairs used are listed in Table S4.

10.1128/mBio.03233-21.9 TABLE S4 Primers used in this study. Download Table S4, DOCX file, 0.02 MB. Copyright © 2022 Mullineaux-Sanders et al. 2022 Mullineaux-Sanders et al. https://creativecommons.org/licenses/by/4.0/ This content is distributed under the terms of the Creative Commons Attribution 4.0 International license.

### Flow cytometry analysis of purified cIEC samples and immune cell populations in colonic tissue.

cIECs were purified as described above. Cells were passed through a 70-μm cell strainer to ensure a single-cell suspension. cIECs were blocked with fluorescence-activated cell sorter (FACS) buffer (2% FBS in PBS) supplemented with Fc block (Miltenyi Biotec) and incubated on ice for 10 min. Samples were then incubated with FACS buffer containing EpCAM-allophycocyanin (APC) (1:100) (clone G8.8; Thermo Fisher Scientific), CD45-fluorescein isothiocyanate (FITC) (1:100) (clone 30-F11; Thermo Fisher Scientific), and phycoerythrin (PE) I-A/I-E (1:100) (clone M5/114.15.2; BioLegend) for 40 min on ice (the gating strategy is shown in Fig. S5A), washed, and fixed in 1% paraformaldehyde in PBS prior to analysis.

10.1128/mBio.03233-21.5 FIG S5 Flow cytometry gating strategies (see Materials and Methods). Flow cytometry gating strategies for cIECs expressing MHCII (A) and colonic immune cells (B) are shown. Download FIG S5, PDF file, 0.9 MB. Copyright © 2022 Mullineaux-Sanders et al. 2022 Mullineaux-Sanders et al. https://creativecommons.org/licenses/by/4.0/ This content is distributed under the terms of the Creative Commons Attribution 4.0 International license.

The colonic tissue remaining after cIEC purification was washed in RPMI 1640 to remove any EDTA left from the cIEC extraction buffer, sliced, and transferred into a c-Mac tube containing 4 mL of complete RPMI medium supplemented with Liberase TM (0.13-mg/mL final concentration; Roche) and DNase I (10 μg/mL; Sigma-Aldrich). A first mild homogenization step was carried out using a gentleMACS dissociator (Miltenyi Biotec), followed by enzymatic digestion for 30 min on a shaker at 37°C. A total of 10 mM EDTA was added to the samples to inhibit enzymatic activity before performing a second homogenization step with the gentleMACS dissociator. The cells were then filtered through a 70-μm cell strainer, centrifuged at 4°C (2,200 rpm for 10 min), and resuspended in 300 μL of complete RPMI medium. From here onward, steps were carried out on ice to preserve cell integrity. For staining, 100 μL from each sample (∼5 × 10^5^ cells) was added to each well of a 96-well V-bottom plate; leftover cells were used for fluorescence-minus-one (FMO) and unstained controls. Dead cells were routinely excluded with Zombie Aqua fixable dead-cell stain (Thermo Fisher Scientific). Single-cell suspensions were incubated with Fc block (BioLegend) in FACS buffer, followed by staining at 4°C in the dark with the following conjugated antibodies in FACS buffer: CD4-BV421 (1:50) (clone RM4-5), CD64-BV605 (1:100) (clone X54-5/7.1), B220-BV711 (1:200) (clone RA3-6B2), CD3-peridinin chlorophyll protein (PerCP)-Cy5.5 (1:100) (clone 145-2C11), Ly6G-PE (1:400) (clone 1A8), and CD8-APC-Cy7 (1:100) (clone 53-6.7) (all from BioLegend) and CD45-FITC, F4/80-PE-Cy5 (1:200) (clone BM8), CD11c-PE-Cy7 (1:300) (clone N418), and CD11b-AF700 (1:400) (clone M1/70) (all from Thermo Fisher Scientific). Briefly, neutrophils were defined as CD11b^+^ Ly6G^+^ cells, monocytes/macrophages were defined as CD11b^+^ F4/80^+^ CD64^+^ cells, conventional dendritic cells (cDCs) were defined as CD11b^+^ CD64^−^ CD11c^+^ cells, B cells were defined as CD45^+^ CD3^−^ B220^+^ cells, and T cells were defined as CD45^+^ B220^−^ CD3^+^ cells (either CD4^+^ or CD8^+^) (the gating strategy is shown in Fig. S5B). Cells were then washed and fixed for 20 min with 1% paraformaldehyde in PBS; after fixing, the cells were kept in the dark at 4°C until analysis. Absolute numbers of immune cells in the sample were calculated using CountBright absolute beads (Thermo Fisher Scientific).

Single-stain controls for compensation were prepared using VersaComp beads (Beckman Coulter) and ArC amine-reactive compensation beads (Thermo Fisher Scientific). Flow cytometry analysis on 50,000 single cells for cIEC analysis and 50,000 live cells for immune cell analysis was performed on a BD LSRFortessa cell analyzer (BD Biosciences). Data were analyzed using FlowJo v10.7.1. Any immune cell samples with fewer than 40% live cells were excluded from further analysis.

### Statistical analysis and data presentation of proteomics data.

Column normalization was used to correct for different protein loading in each channel, and each TMT set was scaled within itself to give relative abundance values. Where proteins were absent in some of the TMT channels, missing values were replaced with the minimum relative abundance value in the TMT set. For each infection time point, Log_2_FC values were calculated compared to the mock-infected mice in each experiment. Log_2_FC values were averaged across the three biological repeats for data representation. Proteins identified in only one biological repeat were excluded from all analyses. 1D enrichment, PCA, and statistical analysis of proteomic data sets were performed in Perseus (version 1.6.12) (49). Proteins significantly changed among all groups were determined by one-way ANOVA with permutation-based FDR correction (FDR of <0.05) for multiple testing, performed on the relative abundance values. For comparison of individual groups, Tukey’s honestly significant difference (HSD) posttest was applied to proteins considered significantly changed by one-way ANOVA (FDR of <0.05). The “Hallmarks” annotation was extracted from the msigdb package in R (50). 1D enrichment values in Fig. 1D represent the average enrichment scores, where the process was considered significantly enriched in at least 2 out of 3 biological repeats. Heat maps were generated and hierarchal clustering was performed using Morpheus (https://software.broadinstitute.org/morpheus). For comparison of the data reported here with our previously published proteomic data sets from the establishment and early steady-state stages of C. rodentium infection (20, 28), data were normalized using z scores [z score = (Log_2_FC − mean sample Log_2_FC)/standard deviation of the sample Log_2_FC]. The “top changed” proteins at 10 to 20 DPI (presented in Fig. S3A) indicate the proteins with the greatest Log_2_FC when the average Log_2_FC values of time points from 10 to 20 DPI of all proteins considered significantly changed (FDR of <0.05 by one-way ANOVA) are ranked from highest to lowest in terms of the absolute Log_2_FC.

### Statistical analysis and data presentation of nonproteomic data.

Data were plotted and statistically analyzed in Prism (version 8). Data that passed a Shapiro-Wilk normality test (quantitative reverse-transcription PCR [RT-qPCR] data) or the Kolmogorov-Smirnov normality test (all other data) were analyzed using ordinary or repeated-measures (RM) one-way ANOVA with Dunnett’s posttest for multiple comparisons to a control group or unpaired two-tailed Student’s *t* test, as specified in the figure legends. *P* values of <0.05 were considered significant. Data that did not pass the specified normality test were log transformed, and, if necessary, the robust regression and outlier removal (ROUT) method (where *Q*, which reflects the probability of falsely identifying an outlier, was kept at 1%) was then applied to identify any outliers that were removed prior to statistical analysis. Log-transformed data with outliers removed that did not pass the specified normality test were not subjected to further statistical analysis, as stated in the figure legends. For statistical analysis of IFN-γ ELISA data for colonic explants (Fig. 4K), the data point that fell below the limit of detection (31.25 pg/g tissue) was given an imputed value of 30.

### Data replicates.

For temporal proteomic and qRT-PCR analyses, four biological repeats (three biological repeats for proteomics analysis and one biological repeat for qRT-PCR analysis), each using five mice/group (time point), were performed. Histopathological and immunofluorescence analyses of the distal colon at 10 to 48 DPI were performed on the same mice as the ones used for proteomics and qRT-PCR analyses. For all other experiments, biological replicates are detailed in the figure legends.

### Data availability.

The mass spectrometry proteomics data have been deposited to the ProteomeXchange Consortium via the PRIDE (51) partner repository with the data set accession number PXD022230.

## References

[1] Haber AL, Biton M, Rogel N, Herbst RH, Shekhar K, Smillie C, Burgin G, Delorey TM, Howitt MR, Katz Y, Tirosh I, Beyaz S, Dionne D, Zhang M, Raychowdhury R, Garrett WS, Rozenblatt-Rosen O, Shi HN, Yilmaz O, Xavier RJ, Regev A. 2017. A single-cell survey of the small intestinal epithelium. Nature 551:333–339. doi:10.1038/nature24489. 29144463 PMC6022292

[2] Peterson LW, Artis D. 2014. Intestinal epithelial cells: regulators of barrier function and immune homeostasis. Nat Rev Immunol 14:141–153. doi:10.1038/nri3608. 24566914

[3] Okumura R, Takeda K. 2017. Roles of intestinal epithelial cells in the maintenance of gut homeostasis. Exp Mol Med 49:e338. doi:10.1038/emm.2017.20. 28546564 PMC5454438

[4] Fukata M, Arditi M. 2013. The role of pattern recognition receptors in intestinal inflammation. Mucosal Immunol 6:451–463. doi:10.1038/mi.2013.13. 23515136 PMC3730813

[5] Zheng Y, Valdez PA, Danilenko DM, Hu Y, Sa SM, Gong Q, Abbas AR, Modrusan Z, Ghilardi N, De Sauvage FJ, Ouyang W. 2008. Interleukin-22 mediates early host defense against attaching and effacing bacterial pathogens. Nat Med 14:282–289. doi:10.1038/nm1720. 18264109

[6] Odenwald MA, Turner JR. 2017. The intestinal epithelial barrier: a therapeutic target? Nat Rev Gastroenterol Hepatol 14:9–21. doi:10.1038/nrgastro.2016.169. 27848962 PMC5554468

[7] Barker N, Van Es JH, Kuipers J, Kujala P, Van Den Born M, Cozijnsen M, Haegebarth A, Korving J, Begthel H, Peters PJ, Clevers H. 2007. Identification of stem cells in small intestine and colon by marker gene Lgr5. Nature 449:1003–1007. doi:10.1038/nature06196. 17934449

[8] Sasaki N, Sachs N, Wiebrands K, Ellenbroek SIJ, Fumagalli A, Lyubimova A, Begthel H, van den Born M, van Es JH, Karthaus WR, Li VSW, López-Iglesias C, Peters PJ, van Rheenen J, van Oudenaarden A, Clevers H. 2016. Reg4+ deep crypt secretory cells function as epithelial niche for Lgr5+ stem cells in colon. Proc Natl Acad Sci USA 113:E5399–E5407. doi:10.1073/pnas.1607327113. 27573849 PMC5027439

[9] Humphries A, Wright NA. 2008. Colonic crypt organization and tumorigenesis. Nat Rev Cancer 8:415–424. doi:10.1038/nrc2392. 18480839

[10] Zheng D, Liwinski T, Elinav E. 2020. Interaction between microbiota and immunity in health and disease. Cell Res 30:492–506. doi:10.1038/s41422-020-0332-7. 32433595 PMC7264227

[11] Mullineaux-Sanders C, Sanchez-Garrido J, Hopkins EGD, Shenoy AR, Barry R, Frankel G. 2019. *Citrobacter rodentium*-host-microbiota interactions: immunity, bioenergetics and metabolism. Nat Rev Microbiol 17:701–715. doi:10.1038/s41579-019-0252-z. 31541196

[12] Slater SL, Sågfors AM, Pollard DJ, Ruano-Gallego D, Frankel G. 2018. The type III secretion system of pathogenic *Escherichia coli*, p 51–72. *In* Frankel G, Ron EZ (ed), Escherichia coli, a versatile pathogen. Springer International Publishing, Cham, Switzerland. 10.1007/82_2018_116 30088147

[13] Crepin VF, Habibzay M, Glegola-Madejska I, Guenot M, Collins JW, Frankel G. 2015. Tir triggers expression of CXCL1 in enterocytes and neutrophil recruitment during Citrobacter rodentium infection. Infect Immun 83:3342–3354. doi:10.1128/IAI.00291-15. 26077760 PMC4534649

[14] Nadler C, Baruch K, Kobi S, Mills E, Haviv G, Farago M, Alkalay I, Bartfeld S, Meyer TF, Ben-Neriah Y, Rosenshine I. 2010. The type III secretion effector NleE inhibits NF-κB activation. PLoS Pathog 6:e1000743. doi:10.1371/journal.ppat.1000743. 20126447 PMC2813277

[15] Baruch K, Gur-Arie L, Nadler C, Koby S, Yerushalmi G, Ben-Neriah Y, Yogev O, Shaulian E, Guttman C, Zarivach R, Rosenshine I. 2011. Metalloprotease type III effectors that specifically cleave JNK and NF-κB. EMBO J 30:221–231. doi:10.1038/emboj.2010.297. 21113130 PMC3020117

[16] Ruano-Gallego D, Sanchez-Garrido J, Kozik Z, Núñez-Berrueco E, Cepeda-Molero M, Mullineaux-Sanders C, Clark JN-B, Slater SL, Wagner N, Glegola-Madejska I, Roumeliotis TI, Pupko T, Fernández LÁ, Rodríguez-Patón A, Choudhary JS, Frankel G. 2021. Type III secretion system effectors form robust and flexible intracellular virulence networks. Science 371:eabc9531. doi:10.1126/science.abc9531. 33707240

[17] Thanabalasuriar A, Koutsouris A, Weflen A, Mimee M, Hecht G, Gruenheid S. 2010. The bacterial virulence factor NleA is required for the disruption of intestinal tight junctions by enteropathogenic *Escherichia coli*. Cell Microbiol 12:31–41. doi:10.1111/j.1462-5822.2009.01376.x. 19712078 PMC2850276

[18] Xia X, Liu Y, Hodgson A, Xu D, Guo W, Yu H, She W, Zhou C, Lan L, Fu K, Vallance BA, Wan F. 2019. EspF is crucial for *Citrobacter rodentium*-induced tight junction disruption and lethality in immunocompromised animals. PLoS Pathog 15:e1007898. doi:10.1371/journal.ppat.1007898. 31251784 PMC6623547

[19] Wiles S, Clare S, Harker J, Huett A, Young D, Dougan G, Frankel G. 2004. Organ specificity, colonization and clearance dynamics *in vivo* following oral challenges with the murine pathogen *Citrobacter rodentium*. Cell Microbiol 6:963–972. doi:10.1111/j.1462-5822.2004.00414.x. 15339271

[20] Hopkins EGD, Roumeliotis TI, Mullineaux-Sanders C, Choudhary JS, Frankel G. 2019. Intestinal epithelial cells and the microbiome undergo swift reprogramming at the inception of colonic *Citrobacter rodentium* infection. mBio 10:e00062-19. doi:10.1128/mBio.00062-19. 30940698 PMC6445932

[21] Crepin VF, Collins JW, Habibzay M, Frankel G. 2016. *Citrobacter rodentium* mouse model of bacterial infection. Nat Protoc 11:1851–1876. doi:10.1038/nprot.2016.100. 27606775

[22] Satoh-Takayama N, Vosshenrich CAJ, Lesjean-Pottier S, Sawa S, Lochner M, Rattis F, Mention JJ, Thiam K, Cerf-Bensussan N, Mandelboim O, Eberl G, Di Santo JP. 2008. Microbial flora drives interleukin 22 production in intestinal NKp46+ cells that provide innate mucosal immune defense. Immunity 29:958–970. doi:10.1016/j.immuni.2008.11.001. 19084435

[23] Backert I, Koralov SB, Wirtz S, Kitowski V, Billmeier U, Martini E, Hofmann K, Hildner K, Wittkopf N, Brecht K, Waldner M, Rajewsky K, Neurath MF, Becker C, Neufert C. 2014. STAT3 activation in Th17 and Th22 cells controls IL-22-mediated epithelial host defense during infectious colitis. J Immunol 193:3779–3791. doi:10.4049/jimmunol.1303076. 25187663

[24] Jarade A, Di Santo JP, Serafini N. 2021. Group 3 innate lymphoid cells mediate host defense against attaching and effacing pathogens. Curr Opin Microbiol 63:83–91. doi:10.1016/j.mib.2021.06.005. 34274597

[25] Simmons CP, Goncalves NS, Ghaem-Maghami M, Bajaj-Elliott M, Clare S, Neves B, Frankel G, Dougan G, MacDonald TT. 2002. Impaired resistance and enhanced pathology during infection with a noninvasive, attaching-effacing enteric bacterial pathogen, *Citrobacter rodentium*, in mice lacking IL-12 or IFN-γ. J Immunol 168:1804–1812. doi:10.4049/jimmunol.168.4.1804. 11823513

[26] Silberger D, Zindl C, Moseley C, Maynard C, Vallance B, Zajac A, Weaver C. 2015. CD4+ T cell-derived interleukin-21 is required for the efficient clearance of *Citrobacter rodentium* (MUC5P.744). J Immunol 194:138.2.

[27] Cho H, Jaime H, de Oliveira RP, Kang B, Spolski R, Vaziri T, Myers TG, Thovarai V, Shen Z, Fox JG, Leonard WJ, Kelsall BL. 2019. Defective IgA response to atypical intestinal commensals in IL-21 receptor deficiency reshapes immune cell homeostasis and mucosal immunity. Mucosal Immunol 12:85–96. doi:10.1038/s41385-018-0056-x. 30087442 PMC6301133

[28] Berger CN, Crepin VF, Roumeliotis TI, Wright JC, Carson D, Pevsner-Fischer M, Furniss RCD, Dougan G, Dori-Bachash M, Yu L, Clements A, Collins JW, Elinav E, Larrouy-Maumus GJ, Choudhary JS, Frankel G. 2017. *Citrobacter rodentium* subverts ATP flux and cholesterol homeostasis in intestinal epithelial cells *in vivo*. Cell Metab 26:738–752.e6. doi:10.1016/j.cmet.2017.09.003. 28988824 PMC5695859

[29] Chan JM, Bhinder G, Sham HP, Ryz N, Huang T, Bergstrom KS, Vallance BA. 2013. CD4^+^ T cells drive goblet cell depletion during *Citrobacter rodentium* infection. Infect Immun 81:4649–4658. doi:10.1128/IAI.00655-13. 24101690 PMC3837981

[30] Parikh K, Antanaviciute A, Fawkner-Corbett D, Jagielowicz M, Aulicino A, Lagerholm C, Davis S, Kinchen J, Chen HH, Alham NK, Ashley N, Johnson E, Hublitz P, Bao L, Lukomska J, Andev RS, Björklund E, Kessler BM, Fischer R, Goldin R, Koohy H, Simmons A. 2019. Colonic epithelial cell diversity in health and inflammatory bowel disease. Nature 567:49–55. doi:10.1038/s41586-019-0992-y. 30814735

[31] Bishu S, Hou G, El Zaatari M, Bishu SR, Popke D, Zhang M, Grasberger H, Zou W, Stidham RW, Higgins PDR, Spence JR, Kamada N, Kao JY. 2019. *Citrobacter rodentium* induces tissue-resident memory CD4^+^ T cells. Infect Immun 87:e00295-19. doi:10.1128/IAI.00295-19. 31061145 PMC6589064

[32] Roche PA, Furuta K. 2015. The ins and outs of MHC class II-mediated antigen processing and presentation. Nat Rev Immunol 15:203–216. doi:10.1038/nri3818. 25720354 PMC6314495

[33] Crotty S. 2019. T follicular helper cell biology: a decade of discovery and diseases. Immunity 50:1132–1148. doi:10.1016/j.immuni.2019.04.011. 31117010 PMC6532429

[34] Aiken KD, Kisslinger JA, Roth KA. 1994. Immunohistochemical studies indicate multiple enteroendocrine cell differentiation pathways in the mouse proximal small intestine. Dev Dyn 201:63–70. doi:10.1002/aja.1002010107. 7803848

[35] Gunawardene AR, Corfe BM, Staton CA. 2011. Classification and functions of enteroendocrine cells of the lower gastrointestinal tract. Int J Exp Pathol 92:219–231. doi:10.1111/j.1365-2613.2011.00767.x. 21518048 PMC3144510

[36] Kumar A, Russell RM, Pifer R, Menezes-Garcia Z, Cuesta S, Narayanan S, MacMillan JB, Sperandio V. 2020. The serotonin neurotransmitter modulates virulence of enteric pathogens. Cell Host Microbe 28:41–53.e8. doi:10.1016/j.chom.2020.05.004. 32521224 PMC7351610

[37] McElrath C, Espinosa V, Lin J-D, Peng J, Sridhar R, Dutta O, Tseng H-C, Smirnov SV, Risman H, Sandoval MJ, Davra V, Chang Y-J, Pollack BP, Birge RB, Galan M, Rivera A, Durbin JE, Kotenko SV. 2021. Critical role of interferons in gastrointestinal injury repair. Nat Commun 12:2624. doi:10.1038/s41467-021-22928-0. 33976143 PMC8113246

[38] Pearson JS, Giogha C, Mühlen S, Nachbur U, Pham CLL, Zhang Y, Hildebrand JM, Oates CV, Lung TWF, Ingle D, Dagley LF, Bankovacki A, Petrie EJ, Schroeder GN, Crepin VF, Frankel G, Masters SL, Vince J, Murphy JM, Sunde M, Webb AI, Silke J, Hartland EL. 2017. EspL is a bacterial cysteine protease effector that cleaves RHIM proteins to block necroptosis and inflammation. Nat Microbiol 2:16258. doi:10.1038/nmicrobiol.2016.258. 28085133 PMC7613272

[39] Hopfner KP, Hornung V. 2020. Molecular mechanisms and cellular functions of cGAS-STING signalling. Nat Rev Mol Cell Biol 21:501–521. doi:10.1038/s41580-020-0244-x. 32424334

[40] Martin PK, Marchiando A, Xu R, Rudensky E, Yeung F, Schuster SL, Kernbauer E, Cadwell K. 2018. Autophagy proteins suppress protective type I interferon signalling in response to the murine gut microbiota. Nat Microbiol 3:1131–1141. doi:10.1038/s41564-018-0229-0. 30202015 PMC6179362

[41] Vanaja SK, Rathinam VAK, Atianand MK, Kalantari P, Skehan B, Fitzgerald KA, Leong JM. 2014. Bacterial RNA:DNA hybrids are activators of the NLRP3 inflammasome. Proc Natl Acad Sci USA 111:7765–7770. doi:10.1073/pnas.1400075111. 24828532 PMC4040571

[42] Heuberger C, Pott J, Maloy KJ. 2021. Why do intestinal epithelial cells express MHC class II? Immunology 162:357–367. doi:10.1111/imm.13270. 32966619 PMC7968399

[43] Caballero-Flores G, Sakamoto K, Zeng MY, Wang Y, Hakim J, Matus-Acuña V, Inohara N, Núñez G. 2019. Maternal immunization confers protection to the offspring against an attaching and effacing pathogen through delivery of IgG in breast milk. Cell Host Microbe 25:313–323.e4. doi:10.1016/j.chom.2018.12.015. 30686564 PMC6375740

[44] Biton M, Haber AL, Rogel N, Burgin G, Beyaz S, Schnell A, Ashenberg O, Su C-W, Smillie C, Shekhar K, Chen Z, Wu C, Ordovas-Montanes J, Alvarez D, Herbst RH, Zhang M, Tirosh I, Dionne D, Nguyen LT, Xifaras ME, Shalek AK, von Andrian UH, Graham DB, Rozenblatt-Rosen O, Shi HN, Kuchroo V, Yilmaz OH, Regev A, Xavier RJ. 2018. T helper cell cytokines modulate intestinal stem cell renewal and differentiation. Cell 175:1307–1320.e22. doi:10.1016/j.cell.2018.10.008. 30392957 PMC6239889

[45] Thelemann C, Eren RO, Coutaz M, Brasseit J, Bouzourene H, Rosa M, Duval A, Lavanchy C, Mack V, Mueller C, Reith W, Acha-Orbea H. 2014. Interferon-γ induces expression of MHC class II on intestinal epithelial cells and protects mice from colitis. PLoS One 9:e86844. doi:10.1371/journal.pone.0086844. 24489792 PMC3904943

[46] Schauer DB, Falkow S. 1993. Attaching and effacing locus of a *Citrobacter freundii* biotype that causes transmissible murine colonic hyperplasia. Infect Immun 61:2486–2492. doi:10.1128/iai.61.6.2486-2492.1993. 8500884 PMC280873

[47] Petty NK, Bulgin R, Crepin VF, Cerdeño-Tárraga AM, Schroeder GN, Quail MA, Lennard N, Corton C, Barron A, Clark L, Toribio AL, Parkhill J, Dougan G, Frankel G, Thomson NR. 2010. The *Citrobacter rodentium* genome sequence reveals convergent evolution with human pathogenic *Escherichia coli*. J Bacteriol 192:525–538. doi:10.1128/JB.01144-09. 19897651 PMC2805327

[48] Livak KJ, Schmittgen TD. 2001. Analysis of relative gene expression data using real-time quantitative PCR and the 2−ΔΔCT method. Methods 25:402–408. doi:10.1006/meth.2001.1262. 11846609

[49] Tyanova S, Temu T, Sinitcyn P, Carlson A, Hein MY, Geiger T, Mann M, Cox J. 2016. The Perseus computational platform for comprehensive analysis of (prote)omics data. Nat Methods 13:731–740. doi:10.1038/nmeth.3901. 27348712

[50] Subramanian A, Tamayo P, Mootha VK, Mukherjee S, Ebert BL, Gillette MA, Paulovich A, Pomeroy SL, Golub TR, Lander ES, Mesirov JP. 2005. Gene set enrichment analysis: a knowledge-based approach for interpreting genome-wide expression profiles. Proc Natl Acad Sci USA 102:15545–15550. doi:10.1073/pnas.0506580102. 16199517 PMC1239896

[51] Perez-Riverol Y, Csordas A, Bai J, Bernal-Llinares M, Hewapathirana S, Kundu DJ, Inuganti A, Griss J, Mayer G, Eisenacher M, Pérez E, Uszkoreit J, Pfeuffer J, Sachsenberg T, Yilmaz Ş, Tiwary S, Cox J, Audain E, Walzer M, Jarnuczak AF, Ternent T, Brazma A, Vizcaíno JA. 2019. The PRIDE database and related tools and resources in 2019: improving support for quantification data. Nucleic Acids Res 47:D442–D450. doi:10.1093/nar/gky1106. 30395289 PMC6323896

