# Supplementary figures and images for: Citrobacter rodentium Infection Induces Persistent Molecular Changes and Interferon Gamma-Dependent Major Histocompatibility Complex Class II Expression in the Colonic Epithelium
Source: mBio. 2022 Feb 1;13(1):e03233-21. doi: 10.1128/mbio.03233-21 (PMC8805023; doi:10.1128/mbio.03233-21)

Supplementary Figure 1

A

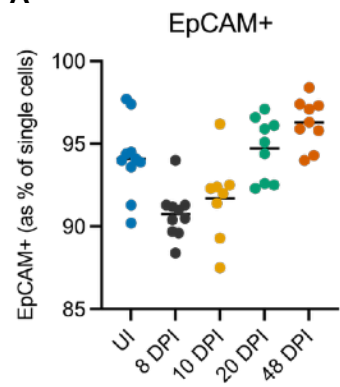

B

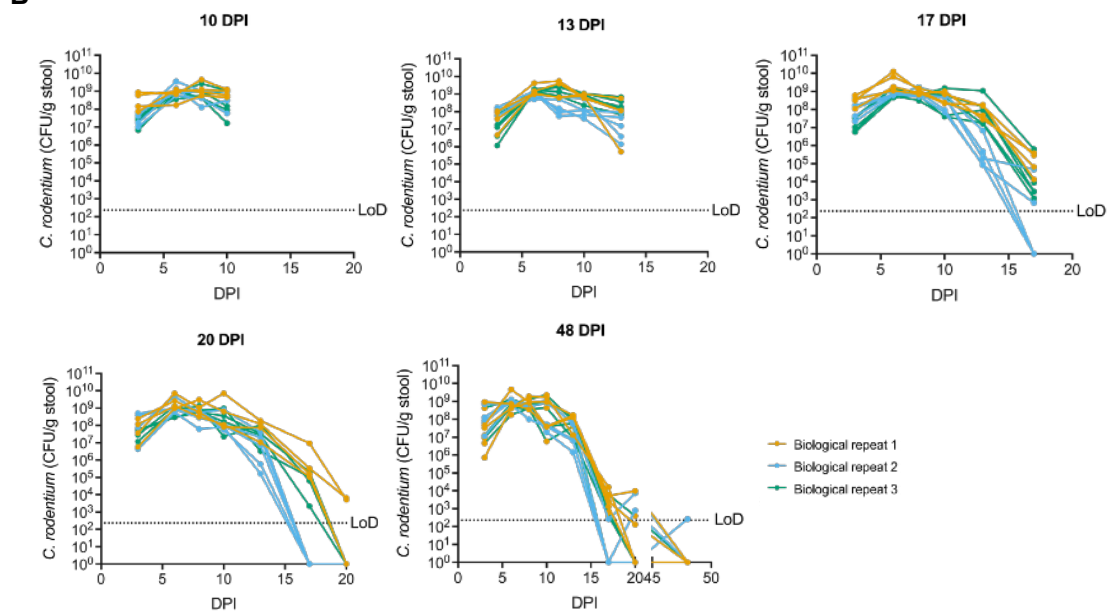

C

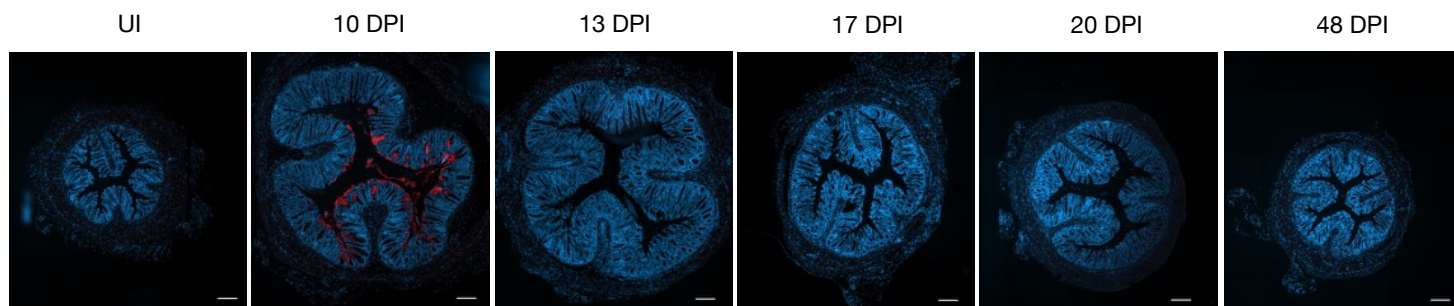

D

|       | 10 DPI | 13 DPI | 17 DPI | 20 DPI | 48 DPI |
|-------|--------|--------|--------|--------|--------|
| Up    | 1900   | 1902   | 1110   | 667    | 37     |
| Down  | 976    | 911    | 689    | 457    | 59     |
| Total | 2876   | 2813   | 1799   | 1124   | 96     |

Supplement: FIG S1 [file mbio.03233-21-sf001.pdf]

Supplementary Figure 2

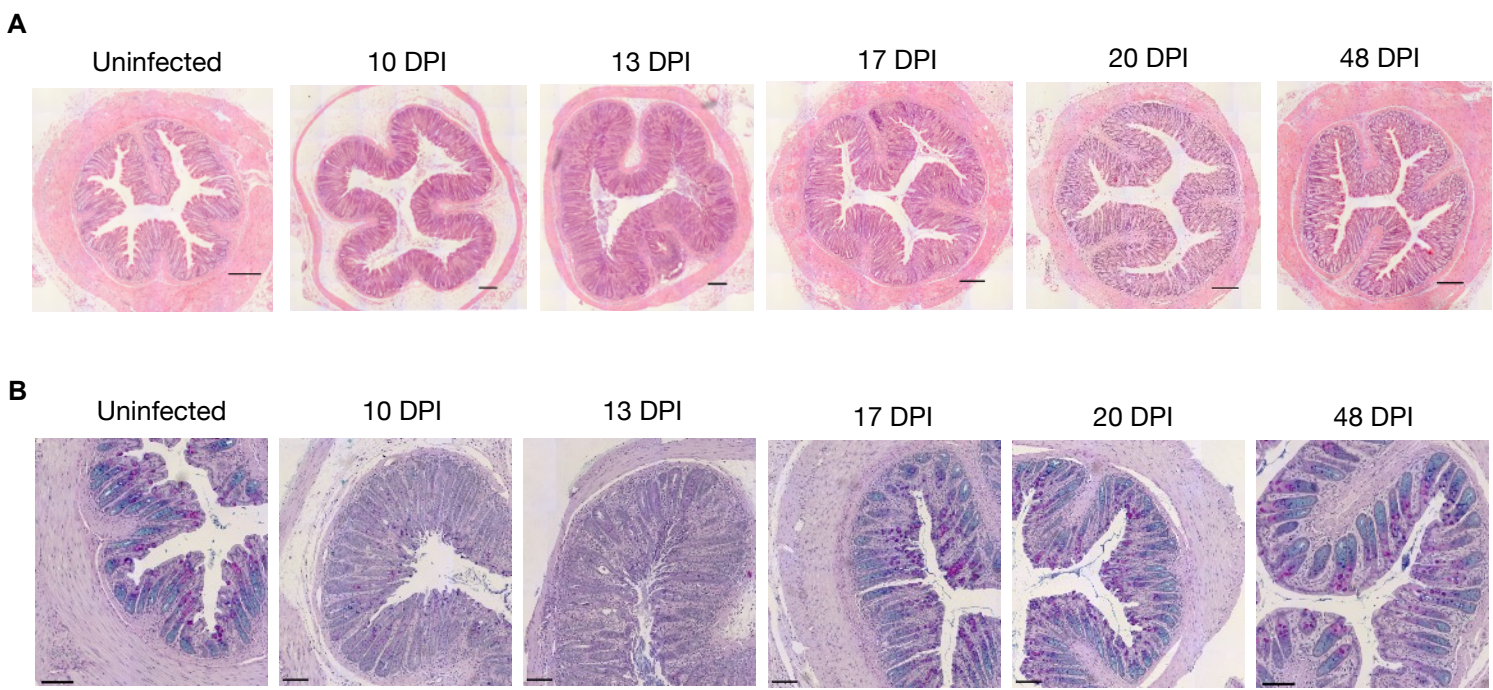

Supplement: FIG S2 [file mbio.03233-21-sf002.pdf]

Supplementary Figure 3

A

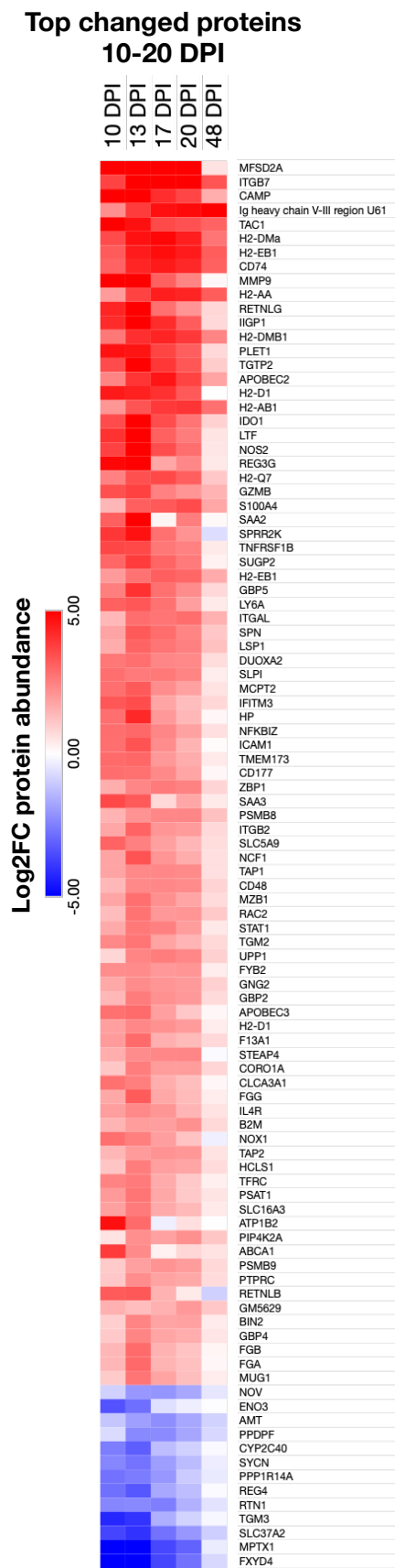

B

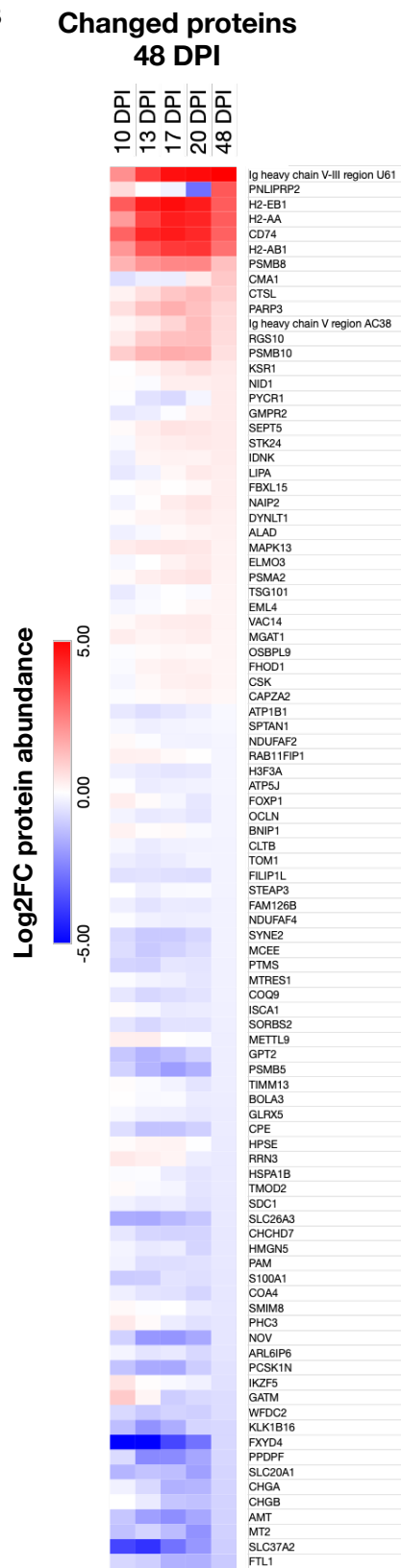

Supplement: FIG S3 [file mbio.03233-21-sf003.pdf]

## Supplementary Figure 4

**A**

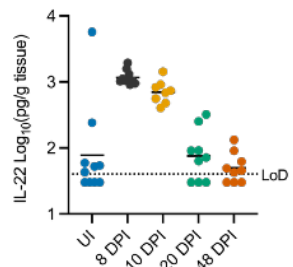

**B**

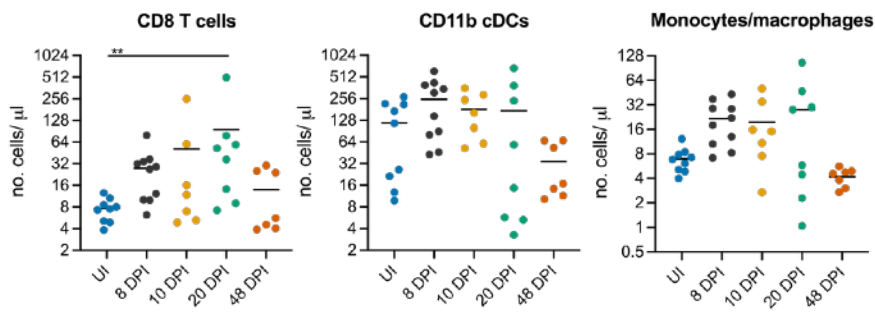

**C**

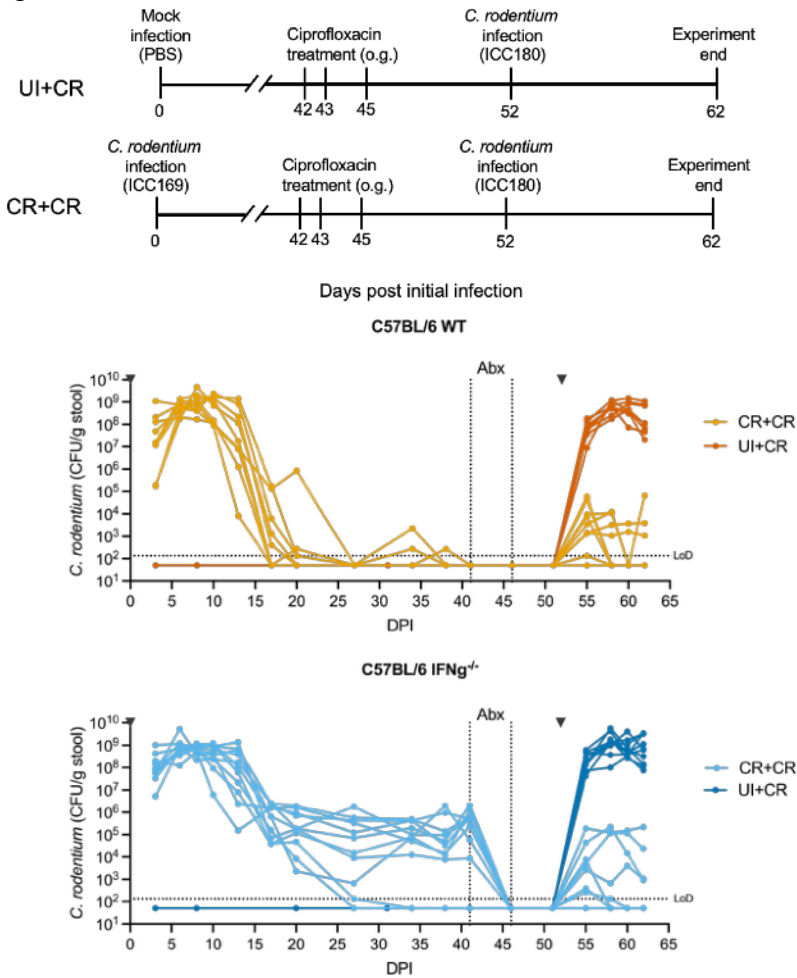

**D**

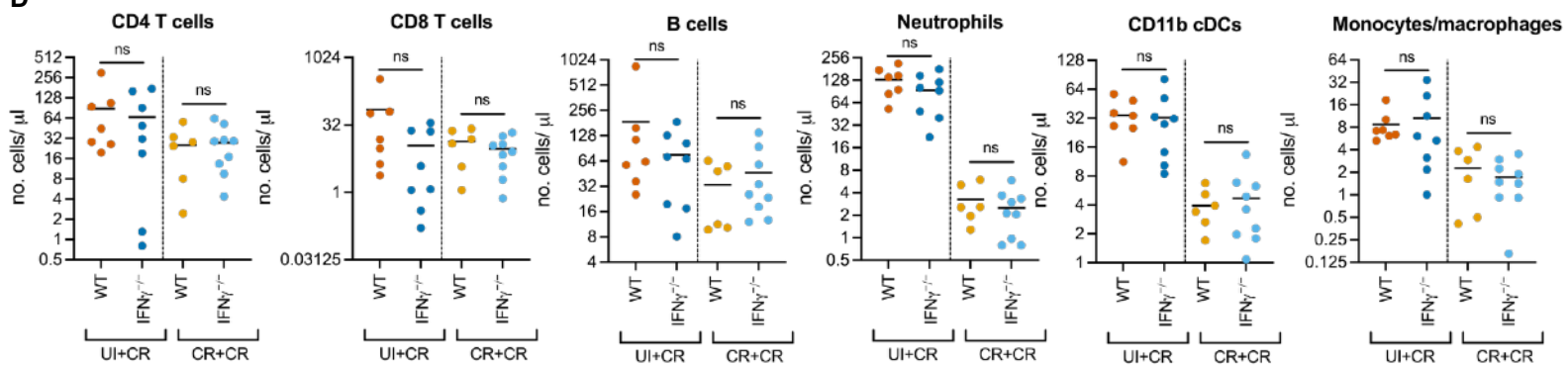

Supplement: FIG S4 [file mbio.03233-21-sf004.pdf]

## Supplementary Figure 5

**A**

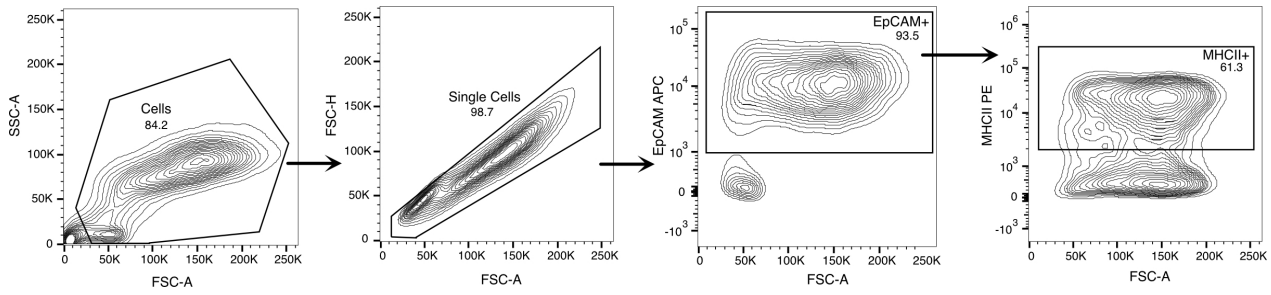

**B**

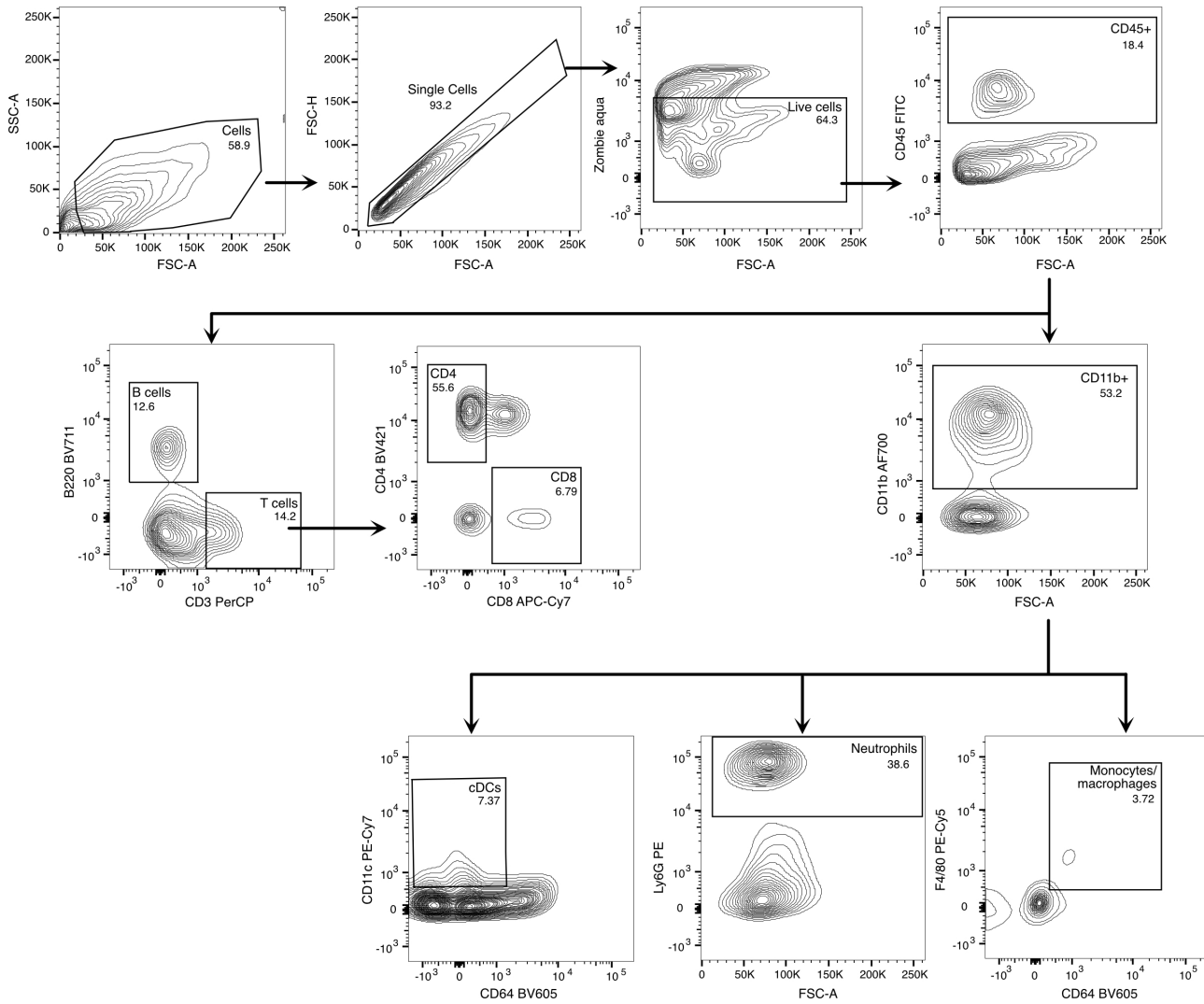

Supplement: FIG S5 [file mbio.03233-21-sf005.pdf]
