# Supplementary material for: Citrobacter rodentium Infection Induces Persistent Molecular Changes and Interferon Gamma-Dependent Major Histocompatibility Complex Class II Expression in the Colonic Epithelium
Source: mBio. 2022 Feb 1;13(1):e03233-21. doi: 10.1128/mbio.03233-21 (PMC8805023; doi:10.1128/mbio.03233-21)
Supplement: TABLE S3 [file mbio.03233-21-st003.docx]

| **Experiment Description** | **Related figures** | **Mouse genotype** | **Group** | **Total mice** | **Mice excluded based on survival criteria** | **Mice excluded based on *C. rodentium* colonisation threshold** | **Mice included in study**  **analysis** |
| --- | --- | --- | --- | --- | --- | --- | --- |
| **Temporal analysis of *C. rodentium*-infected mice** | Fig. 1-3, 4A-J | WT | *C. rodentium* infected | 100 | 1 | 9 | **90** |
|  |  |  | Uninfected | 20 | 0 | N/a | **20** |
|  | Fig. 4K, 5A-C | WT | *C. rodentium* infected | 40 | 0 | 4 | **36** |
|  |  |  | Uninfected | 10 | 0 | N/a | **10** |
| **Temporal analysis of *C. rodentium*-infected IFNγ^-/-^ mice** | Fig. 5D-F | IFNγ^-/-^ | *C. rodentium* infected | 26 | 0 | 1 | **25** |
|  |  |  | Uninfected | 9 | 0 | N/a | **9** |
| ***C. rodentium* rechallenge in WT and IFNγ^-/-^ mice** | Fig. 5G-H | WT | CR+CR | 9 | 0 | 0 ^a^ | **9** |
|  |  |  | UI+CR | 8 | 0 | N/a | **8** |
|  |  | IFNγ^-/-^ | CR+CR | 13 | 1 | 1^a^ | **11** |
|  |  |  | UI+CR | 11 | 1 | N/a | **10** |

**Table S3.**  Table detailing the number of mice excluded from all analysis in each experiment and the reasons behind the exclusion.

^a^ mice excluded for failing to reach the *C. rodentium* colonisation threshold during the first *C. rodentium* infection only.
