# Supplementary material for: Citrobacter rodentium Infection Induces Persistent Molecular Changes and Interferon Gamma-Dependent Major Histocompatibility Complex Class II Expression in the Colonic Epithelium
Source: mBio. 2022 Feb 1;13(1):e03233-21. doi: 10.1128/mbio.03233-21 (PMC8805023; doi:10.1128/mbio.03233-21)
Supplement: TABLE S4 [file mbio.03233-21-st004.docx]

**Table S4. DNA sequences of primers used in this study.**

| **Name** | **Sequence (5’-3’)** |
| --- | --- |
| Gatm_F | GCTTCCTCCCGAAATTCCTGT |
| Gatm_R | CCTCTAAAGGGTCCCATTCGT |
| H2-Aa_F | TGGGCACCATCTTCATCATTC |
| H2-Aa_R | GGTCACCCAGCACACCACTT |
| H2-D1_F | GGGAAACACAGAAAGCCAAG |
| H2-D1_R | AAGTCACAGCCAGACATCTG |
| Psmb6_F | GTACAGAGAAGATCTGATGGCAGGAATC |
| Psmb6_R | GGACTGTCTTACCATCATACCCCCC |
| Psmb9_F | GGTTCCGGAAGCTCCTACAT |
| Psmb9_R | AGAGCCATCTCGGTTCATGG |
| Tap1_F | GGACTTGCCTTGTTCCGAGAG |
| Tap1_R | GCTGCCACATAACTGATAGCGA |
| Ftl1_F | CTCTGGGCGAGTATCTCTTTG |
| Ftl1_R | AGTGGCTTGAGAGGTTCATTC |
| Mt2_F | GCTCCTAGAACTCTTCAAACCG |
| Mt2_R | CAGGAAGTACATTTGCATTGTTTG |
| Chgb_F | TCAAATGCCCTATCCAAGTCC |
| Chgb_R | GCAACCGTACTTCAAACTTCG |
| Aim2_F | TTGTGAATGGGCTGTTTAAAGTC |
| Aim2_R | CCTTCCTCGCACTTTGTTTTG |
| Wfdc2_F | GCTGGCCTCCTACTAGGGTT |
| Wfdc2_R | AACACACAGTCCGTAATTGGT |
| Ido1_F | CAATCAAAGCAATCCCCACTG |
| Ido1_R | AAAACGTGTCTGGGTCCAC |
| Tmem173_F | AGCGGAAGTCTCTGCAGTCT |
| Tmem173_R | GGAGCCCTGGTAAGATCAAC |
| Zbp1_F | TGTTGACTTGAGCACAGGAG |
| Zbp1_R | TTCAGGCGGTAAAGGACTTG |
| Gapdh_F | TCAACAGCAACTCCCACTCTTCCA |
| Gapdh_R | ACCCTGTTGCTGTAGCCGTATTCA |
